# Massive Parallel DNA Sequencing of Patients with Inherited Cardiomyopathies in Cyprus and Suggestion of Digenic or Oligogenic Inheritance

**DOI:** 10.3390/genes15030319

**Published:** 2024-02-28

**Authors:** Constantina Koutsofti, Marios Ioannides, Christiana Polydorou, Gregory Papagregoriou, Apostolos Malatras, George Michael, Irene Hadjiioannou, Stylianos Pieri, Eleni M. Loizidou, Christos Eftychiou, Elias Papasavvas, Theodoros Christophides, Anna Alkelai, Manav Kapoor, Alan R. Shuldiner, Panayiotis Avraamides, Constantinos Deltas

**Affiliations:** 1 Molecular Medicine Research Center, biobank.cy Center of Excellence in Biobanking and Biomedical Research, University of Cyprus, Nicosia 2109, Cyprus; koutsofti.constantina@ucy.ac.cy (C.K.); polydorou.christiana@ucy.ac.cy (C.P.); papagregoriou.gregory@ucy.ac.cy (G.P.); malatras.apostolos@ucy.ac.cy (A.M.); michael.giorgos@ucy.ac.cy (G.M.); hadjioannou.eirini@ucy.ac.cy (I.H.); pieri.stylianos@ucy.ac.cy (S.P.); loizidou.eleni@ucy.ac.cy (E.M.L.); 2 Department of Cardiology, Nicosia General Hospital, Nicosia 2029, Cyprus; mariosioannides1969@gmail.com (M.I.); chr6eft@gmail.com (C.E.); tchristophides@doctors.org.uk (T.C.); 3 Platonas Medical Center, Nicosia 1075, Cyprus; ilipapa@cytanet.com.cy; 4 Regeneron Genetics Center, Tarrytown, NY 10591, USA; anna.alkelai@regeneron.com (A.A.); manav.kapoor@regeneron.com (M.K.); alan.shuldiner@regeneron.com (A.R.S.); 5 School of Medicine, University of Cyprus, Nicosia 2109, Cyprus

**Keywords:** cardiogenetics, inherited cardiomyopathies, massive parallel sequencing, digenic or oligogenic inheritance, incomplete penetrance

## Abstract

Inherited cardiomyopathies represent a highly heterogeneous group of cardiac diseases. DNA variants in genes expressed in cardiomyocytes cause a diverse spectrum of cardiomyopathies, ultimately leading to heart failure, arrythmias, and sudden cardiac death. We applied massive parallel DNA sequencing using a 72-gene panel for studying inherited cardiomyopathies. We report on variants in 25 families, where pathogenicity was predicted by different computational approaches, databases, and an in-house filtering analysis. All variants were validated using Sanger sequencing. Familial segregation was tested when possible. We identified 41 different variants in 26 genes. Analytically, we identified fifteen variants previously reported in the Human Gene Mutation Database: twelve mentioned as disease-causing mutations (DM) and three as probable disease-causing mutations (DM?). Additionally, we identified 26 novel variants. We classified the forty-one variants as follows: twenty-eight (68.3%) as variants of uncertain significance, eight (19.5%) as likely pathogenic, and five (12.2%) as pathogenic. We genetically characterized families with a cardiac phenotype. The genetic heterogeneity and the multiplicity of candidate variants are making a definite molecular diagnosis challenging, especially when there is a suspicion of incomplete penetrance or digenic-oligogenic inheritance. This is the first systematic study of inherited cardiac conditions in Cyprus, enabling us to develop a genetic baseline and precision cardiology.

## 1. Introduction

Cardiomyopathies comprise a heterogeneous group of diseases, in which the cardiac muscle is affected in the absence of other pathology for myocardial dysfunction. Based on morphological and functional features, they are subclassified into five forms: hypertrophic cardiomyopathy (HCM), dilated cardiomyopathy (DCM), arrhythmogenic right ventricular cardiomyopathy (ARVC), left ventricular non-compaction cardiomyopathy (LVNC), and restrictive cardiomyopathy (RCM). The common clinical manifestations among the different cardiomyopathies include arrhythmias, heart failure, and sudden cardiac death [1]. The cause of cardiomyopathies may be familial/genetic or non-familial/non-genetic. In the case of familial (inherited) cardiomyopathies, there is a high incentive to identify pathogenic variants in disease genes in affected individuals to enable a subsequent cascade screening of all the at-risk family members.

Over the last decades, many genetic studies strived to define the genetic etiology of inherited cardiomyopathies and, therefore, many pathogenic or likely pathogenic variants have been identified. Most of these variants are in genes encoding mainly sarcomeric, Z-disc, desmosomal, cytoskeletal, and nuclear envelope proteins [2].

In 1990, the first pathogenic variant (p.Arg403Gln) in the *MYH7* gene was discovered in patients with familial HCM [3], and since then, hundreds of pathogenic variants have been identified in all genes encoding proteins of the sarcomere. For this reason, HCM is frequently called a “disease of the sarcomere” [4]. The most frequently mutated genes in patients with HCM are *MYH7* and *MYBCP3*, which encode the cardiac myosin heavy chain-β and the cardiac myosin binding protein C, respectively. Most of the reported variants (>50%) in HCM have been detected in these two genes [5]. 

In 2000, pathogenic variants in genes encoding sarcomeric proteins have been also detected in familial DCM [6]. One of the most frequently affected genes in familial DCM is *TTN*, which encodes the largest protein in the human genome, titin, an essential component of the sarcomere. Likely pathogenic or pathogenic variants in *TTN* were identified in 30% of cases of familial DCM [7]. In contrast to HCM, pathogenic variants in a wide range of genes can cause DCM. Apart from the variants in genes encoding sarcomeric proteins, variants in genes encoding non-sarcomeric proteins (cytoskeletal, desmosomal, nuclear envelope, and transcriptional cofactor proteins) have been shown to cause DCM [8]. 

ARVC is mainly caused by variants in genes encoding desmosomal proteins, and, based on this fact, it is frequently described as a “disease of the desmosome”. The most commonly mutated desmosomal genes are *PKP2* (10–45%), *DSP* (10–45%), *DSG2* (7–10%), and *DSC2* (2%), encoding the plakophilin-2, desmoplakin, desmoglein-2, and desmocollin-2 proteins, respectively [9,10,11]. In addition, non-desmosomal genes, such as *TMEM43*, *TGF-β3*, and *RYR2*, which encode the transmembrane protein 43, transforming growth factor β3, and ryanodine receptor 2, respectively, were shown to cause ARVC [12].

In LVNC, various unrelated genes have been reported to be potential disease causes. The main genes are those encoding sarcomeric proteins, including *MYH7*, *MYBPC3*, *TTN*, *TNNT2*, *ACTC*, and *TPM1* [13]. In some cases, (likely) pathogenic variants have been also identified, for example, in the *TAZ* gene encoding tafazzin, a mitochondrial membrane protein, or in the *LMNA* gene, which encodes lamin, a nuclear envelope protein [14].

The disease-causing genes in RCM overlap with those of HCM, DCM, and LVNC. Specifically, recent studies have reported that the potential genes are *TTN*, *MYH7*, *MYBPC3*, *MYL2*, *TNNC1*, *TNNI3*, *TNNT2*, *TPM1*, *DES*, *LMNA*, and *FLNC.* However, most disease-causing variants have been identified in sarcomere protein genes [15,16,17].

Based on the above, inherited cardiomyopathies are genetically highly heterogeneous; therefore, it is important to sequence multiple genes in search of candidate variants, which is difficult using older, traditional technologies. The genetic and phenotypic overlap observed among the different types of cardiomyopathies adds further complexity and necessitates testing multiple target genes, especially when the clinical diagnosis is unclear.

Currently, the advances made with modern massive parallel sequencing (MPS) technologies, also referred to as next generation sequencing (NGS) technologies, have significantly improved genetic investigations and enabled the effective targeted treatment of patients and family members faster and cheaper [18]. Additionally, they contributed to better clinical management, thereby leading to improvements in the clinical diagnosis and prognosis, clinical surveillance of carriers, and treatment approach [19].

Even though inherited cardiomyopathies constitute a relatively frequent cause of a heart condition, with an estimated prevalence of 1/250 globally, including sudden cardiac death, to our knowledge, in Cyprus, there has not been a systematic genetic investigation of patients in routine clinical practice. At biobank.cy, a Center of Excellence in Biobanking and Biomedical Research, we thought it essential to address this long-due unmet need to the benefit of the patients by offering a genetic diagnosis, even before the onset of symptoms. To this end, we collaborated with the major heart clinic at the Nicosia General Hospital and archived families and DNA to support clinical and genetic investigations. 

## 2. Materials and Methods

### 2.1. Clinical Data and Blood Samples from Patients

The first part of our research studies included 25 probands with a family history of a known phenotype of cardiomyopathy based on clinical signs and evaluations. The diagnosis of the different types of cardiomyopathies was made based on the guidelines of the European Society of Cardiology and the American Heart Association. The probands belonged to fourteen families with HCM, seven with DCM, three with ARVC, and one with non-compaction cardiomyopathy (NCM). 

The clinical and laboratory data were obtained from cardiologists at the Department of Cardiology of the Nicosia General Hospital in Cyprus. The most important parameters that were collected include demographic information, medical history, family history/pedigree, electrocardiogram (ECG) test, echocardiogram (echo) test, exercise test, 24-h Holter monitoring, laboratory tests, and/or cardiac magnetic resonance imaging (MRI). All the personal data are archived in the Research Electronic Data Capture (REDCap v13.1.33) database [20] and all family pedigrees are constructed using PhenoTips software v1.4.4 [21]. Blood samples from the proband and his or her family members were collected and processed in the Biobank of our center.

The project was approved by the Cyprus National Bioethics Committee and all participants gave their informed signed consent.

### 2.2. Targeted Massive Parallel DNA Sequencing

**A.**  **Probands with cardiomyopathies**

Genomic DNA was isolated from peripheral blood leukocytes using a salting out procedure [22]. Then, the DNA sample of each proband was analyzed by NGS using a panel of 72 genes (Table 1), variants in which are known to cause inherited cardiac diseases. The panel includes the most frequently mutated genes but also other less frequently affected genes (Table S1).

Our custom Ion Ampliseq panel was designed through the Ion Ampliseq Designer V.7.4.8.3 tool (Ion Torrent Systems Inc, Gilford, NH, USA) using human genome 19 (hg19 or GRCh37) as a reference sequence. A total of 2995 amplicons ranging from 125 to 275 bp in length were included, covering 510.94 Kb of the total genomic region. The total coverage of all exons of the 72 genes was 99.54%. The coverage of four genes, *TTN*, *ALPK3*, *FLNC*, and *PRDM16*, was 98.3%, 98.5%, 99.5%, and 99.8%, respectively, and for the remaining 68 genes, it was 100%.

For the sequencing procedure, a library was built by amplifying 10 ng of genomic DNA from each proband using the Ion Ampliseq Library Kit v2.0 (Ion Torrent Systems Inc) according to the manufacturer’s instructions. Each sample was barcoded using the Ion Xpress Barcode Adapters (Ion Torrent Systems Inc) for multiplexing. The amplified libraries were then purified with Agencourt AMPure XP beads (Beckman & Coulter Life Sciences, Indianapolis, IN, USA) and quantified for sequencing with a Qubit 2.0 fluorometer (Thermo Fisher Scientific, Waltham, MA, USA) using the Qubit HS dsDNA Assay kit. Subsequently, each library was diluted to 100 pM, and the same amount of the diluted libraries was pooled in one sequencing reaction. The combined libraries were loaded onto the Ion Chef System (Ion Torrent Systems Inc), where clonal amplification of the libraries was performed on Ion Sphere^TM^ Particles (ISPs) by emulsion PCR. In the end, the Ion Chef System provided a sequencing chip loaded with the enriched ISPs, which was then sequenced on the Ion GeneStudio S5 System (Ion Torrent Systems Inc) according to the manufacturer’s instructions.

**B.**  **Individuals of the Cypriot general population**

Genomic DNA from 100 individuals of a general healthy Cypriot population was also sequenced with the same procedure as that described above for the patients’ samples to create an in-house database of variants.

The samples from another 1000 general Cypriot population controls, the CYPROME cohort, were retrieved from biobank.cy and underwent whole exome sequencing at Regeneron Genetics Center, NY, USA. All samples were prepared for sequencing using a custom automated sample preparation workflow developed at Regeneron. Genomic DNA libraries were created by enzymatically shearing DNA to a mean fragment size of 200 base pairs (New England Biolabs, Ipswich, MA, USA). A common Y-shaped adapter (Integrated DNA Technologies, Coralville, IA, USA) was ligated to all DNA libraries. Unique, asymmetric 10 base pair barcodes were added to the DNA fragments during library amplification with Kapa HiFi Mix (Roche, Basel, CH) to facilitate multiplexed exome capture and sequencing. Equal amounts of sample were pooled prior to overnight exome/genotype capture with the Twist Comprehensive Exome panel (Twist Bioscience, San Fransisco, CA, USA), RGC-developed Twist Diversity SNP panel, and additional spike-ins to boost coverage at selected CHIP sites and to cover the mitochondrial genome; all samples were captured on the same lot of oligos. The captured DNA was PCR amplified and quantified by qPCR. The multiplexed samples were pooled and then sequenced using 75 base pair paired-end reads with two 10 base pair index reads on the Illumina NovaSeq 6000 platform (Illumina, San Diego, CA, USA) on S4 flow cells.

### 2.3. Bioinformatics Analysis of the Next Generation Sequencing Data

**A.**  **Probands with cardiomyopathies**

The raw sequencing data from each patient’s sample were transferred into the Ion Torrent Server (Ion Torrent Systems Inc, Gilford, NH, USA) and analyzed using the Ion Torrent Suite^TM^ Software v5.18.1 (Ion Torrent Systems Inc). The analysis consists of base calling, quality control of the raw data, and sequence alignment against the reference human genome 19 (hg19) and variant calling. Then, the resulting aligned reads (BAM files) were used as input by the Ion Reporter^TM^ Software v5.12.2.0 (Ion Torrent Systems Inc) for storage and annotation.

All analyzed variants were annotated by various available public databases, including NCBI-dbSNP [23] and ClinVar [24]. The minor allele frequency (MAF) was obtained from the 1000 Genomes Project Consortium, as provided by the Ion Reporter^TM^ Software [25]. Furthermore, all coding variants with MAF ≤ 0.03 were annotated manually by the Franklin Genoox platform (https://franklin.genoox.com/ (accessed through January 2024), as well as the Human Gene Mutation Database (HGMD) [26]. The final step of the data analysis was variant filtering, where variant call errors and false positive data were removed after an examination of strand bias (MNP Strand Bias value set at 0.95).

Then, the identification of candidate pathogenic variants was performed with the hierarchical filtering described below.

Filtering strategy:

For quality control, the variants that had <20 total reads and allele reads <10 were excluded.

Afterward, the variants were filtered hierarchically as follows:

1. Functional consequences: missense, nonsense, frameshift, in frame coding indels, and splice site (±2 nucleotides) variants;
2. MAF ≤ 0.03 (per the 1000 Genomes Project Consortium);
3. Classified as pathogenic, likely pathogenic, and variants of uncertain significance (VUS) in the Franklin tool (based on the ACMG criteria).

Criterion number 2 regarding the MAF of ≤0.03, which is more liberal compared to the stricter 0.01% (1/10,000) usually used, was chosen based on previous experience with other rare diseases we studied in Cyprus, as well as because of the total lack of data regarding the frequency of inherited cardiomyopathies in the Cypriot population. Additionally, we wanted to cover the remote likelihood of having more frequent but hypomorphic variants that could act in concert with a second variant in trans, thereby following bi-allelic or digenic inheritance.

All the candidate variants, which arose from the above filtering steps, were visualized in the Integrative Genomics Viewer (IGV).

**B.**  **Individuals of the Cypriot general population**

The raw sequencing data from the DNA samples of the 100 individuals were processed at our centre with the same methods as that of the patients’ samples. Subsequently, the individual volunteer variant call format (VCF) files were merged into one using the bcftools utility suite v1.13 [27] to make the downstream analysis more efficient. Then, this VCF file was annotated using the Variant Effect Predictor (VEP v105.0) [28]. We calculated the allele frequency as follows: We counted the alternative alleles and divided them by the total number of alleles. If multiple alternative alleles were present, we calculated their frequency separately. The number of reference and alternative reads was recorded for each variant in each patient.

The sequencing data in FASTQ format from the 1000 individuals of the general Cypriot population were generated from Illumina image data using the bcl2fastq program (Illumina, San Diego, CA, USA). Following the original quality functional equivalent (OQFE) protocol [29], sequence reads were mapped to GRCh38 references using BWA MEM [30] in an alt-aware manner, read duplicates were marked, and additional per-read tags were added. For exome data, single nucleotide variations (SNV) and short insertion and deletions (indels) were identified using a Parabricks accelerated version of DeepVariant v0.10 with a custom WES model and reported in per-sample genome VCF (gVCF). These exome gVCFs were aggregated with GLnexus v1.4.3 using the pre-configured DeepVariantWES setting [31] into joint-genotyped multi-sample project-level VCF (pVCF), which was converted to bed/bim/fam format using PLINK 1.9 [32].

To capitalize on both datasets even though they were coming from two different technologies, we proceeded to merge them as follows: First, we extracted all 72 cardiomyopathy gene panel variants from the 1000 General population exomes. Subsequently, we uplifted the 100 CM panel from hg19 to GRCh38 assembly, so that both datasets have the same reference version, using CrossMap v0.6.0 [33]. We then merged the VCF files using bcftools tools, and included the allele frequencies for this combined dataset, alongside the alternative allele count, total allele count, and total population count from which the variant was read. The final VCF file was uploaded and further annotated in OpenCRAVAT v2.2.7 [34], which was also used as an advanced search tool to identify variant characteristics.

Finally, it is important to compare the selected variants of the patients with those of the Cypriot general population cohort (from their analyzed NGS data). It is important for these variants to not be present in the general population or at least to have a low allele frequency to be considered as candidate pathogenic variants and processed in the next steps.

### 2.4. Sanger DNA Sequencing 

The candidate pathogenic variants found in the proband’s DNA sample were then validated by Sanger Sequencing. First, the PCR product of interest was placed into an ABI 96-well reaction plate with ExoSAP-IT (Applied Biosystems, Waltham, MA, USA). The plate was incubated for 15 min at 37 °C, followed by enzyme inactivation for 15 min at 80 °C. Subsequently, the cycle sequencing reaction was performed using 3 μL of sequencing buffer, 1 μL of primer (10 pM/μL), 2 μL of BigDye V1.1 (Applied Biosystems) and ddH_2_O up to 20 μL. The reaction was performed in a cycler as follows: 96 °C for 1 min, followed by 29 cycles of 96 °C for 10 s, 50 °C for 5 s, and 60 °C for 4 min.

The next step was the purification of cycle sequencing products using EDTA/EtOH. Following the purification step, 10 μL of HiDi Formamide (Applied Biosystems) was added to each well of the plate. Subsequently, the plate was loaded onto the 3500 Genetic Analyzer (Applied Biosystems). The results were analyzed with the Sequencing Analysis Software v5.2 (Applied Biosystems). The genotype of the region of interest was determined by visualizing the sequencing electropherograms. 

In case the candidate variants were validated in the proband’s sample, the at-risk family members were also examined by Sanger sequencing.

## 3. Results

We herein present our results of testing probands and relatives from a subset of twenty-five families: fourteen with HCM, seven with DCM, three with ARVC, and one with NCM. Familial segregation was not always clear and unequivocal. We report on the genetic findings using a combination of an MPS approach followed by verification with a Sanger sequencing methodology and segregation in the respective families. 

### 3.1. Clinical Data of Patients

Most patients complained of easy fatigue and shortness of breath at presentation. Other symptoms were near fainting or fainting and palpitation. Those with a prior heart evaluation by a primary cardiologist were on treatment for the diagnosed cardiomyopathy. Patients or family members diagnosed with a form of cardiomyopathy received treatment based on current guidelines [35]. If the guidelines indicated an Implantable Cardioverter Defibrillator (ICD) they were referred to the electrophysiology services of our department.

In most patients, there were abnormalities on the electrocardiogram, mainly in terms of ST-T non-specific changes. The diagnosis of cardiomyopathy was established by echocardiographic studies. Cardiac magnetic resonance was useful to confirm the diagnosis in some borderline cases and contributed to the stratification of risk for future arrhythmic events. Based on the medical history, clinical examination and diagnostic exercise tests, 24-h Holter monitoring and electrophysiological studies were performed.

Most family members who shared the putative causative genotype of the proband were clinically asymptomatic, even if they had an abnormal electrocardiogram or abnormal echocardiographic or MRI findings. Some family members complained of irrelevant symptoms (i.e., headache or musculoskeletal pains). We fully investigated genotype positive–phenotype negative patients with the full spectrum of imaging and functional tests to confirm the absence of any clinical findings related to cardiomyopathy.

### 3.2. Genetic Investigations and DNA Mutation Findings

Our sequencing panel included 72 genes, which, according to literature data, can cause a cardiac disease when mutated. On average, in each sequenced proband, 390 DNA variants were identified after the first filtering of validated data by the Ion Torrent NGS technology. These variants were then examined by performing an in silico analysis, as described in the Methods section, using the Franklin Genoox platform (https://franklin.genoox.com/ (accessed through January 2024). Interestingly, our further variant filtering strategy resulted in identifying one to three candidate pathogenic variants per proband, which were validated by Sanger DNA sequencing. Familial segregation was also tested by sequencing when possible.

**Figure 1:**
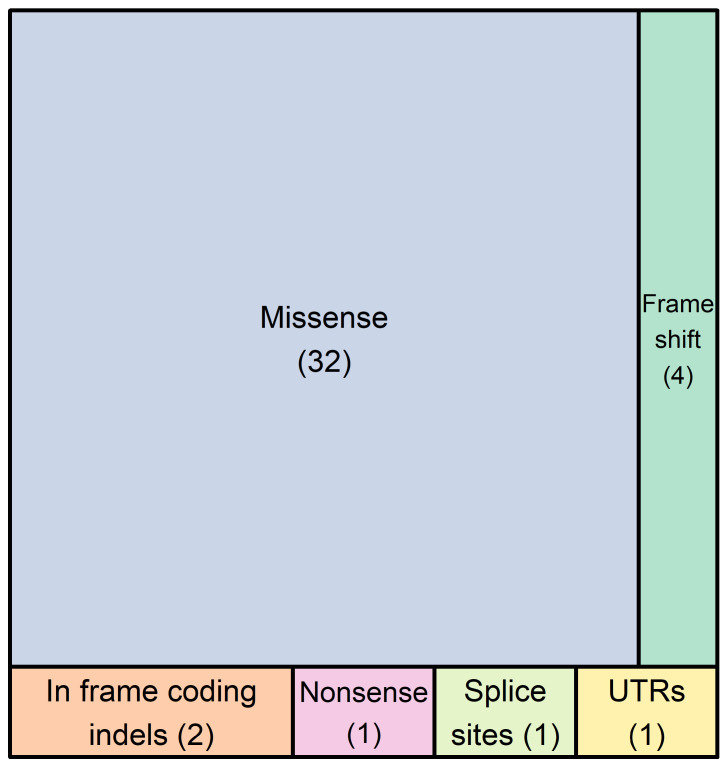
The number of DNA variants found in 26 genes of our 72-gene panel in 25 probands. In total, forty-one different genetic variants were detected, which included thirty-two single nucleotide substitutions, one premature termination codon, four frameshift variants resulting in premature termination codons, two in frame coding indels, one variant affecting the canonical splice site nucleotides, and one variant in the first nucleotide of the 3′-UTR. These variants belong to 26 genes, which are the following: *ALPK3*, *ANK2*, *DES*, *DSC2*, *DSP*, *FLNC*, *KCNQ1*, *LAMA4*, *LAMP2*, *LMNA*, *MYBPC3*, *MYH6*, *MYH7*, *MYL2*, *MYPN*, *NEBL*, *NEXN*, *PKP2*, *PLN*, *RBM20*, *RYR2*, *SCN5A*, *TNNC1*, *TNNI3*, *TTN*, and *VCL*.

In the 25 probands/families tested here, a total of 41 different non-synonymous DNA variants were recorded in 26 genes. The types of genetic variants detected are the following: thirty-two (78%) missense variants, one (2.4%) nonsense variant, four (9.8%) rameshift variants resulting in premature termination codons, two (4.9%) in frame coding indels, one (2.4%) variant affecting the consensus splice site nucleotides, and one (2.4%) variant in the first nucleotide of the 3′-UTR (Figure 1). 

**Figure 2:**
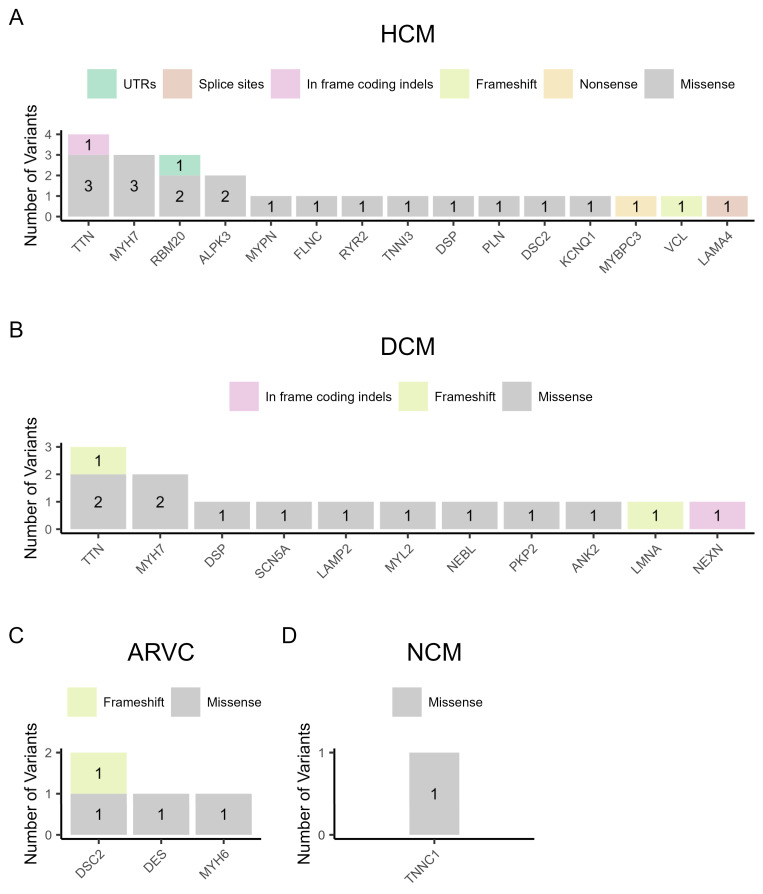
The variant types per gene in the four categories of cardiomyopathy. (**A**) Hypertrophic cardiomyopathy (HCM), (**B**) dilated cardiomyopathy (DCM), (**C**) arrhythmogenic right ventricular cardiomyopathy (ARVC), and (**D**) non-compaction cardiomyopathy (NCM).

Figure 2 shows the distribution of variant types per gene for each cardiomyopathy. Specifically, in the 14 families with HCM, we found twenty-three non-synonymous variants in 15 different genes, which included eighteen missense variants, one nonsense variant in the *MYBPC3* gene, one frameshift variant in the *VCL* gene, one in frame coding indel in the *TTN* gene, one deletion of the first four nucleotides in the donor spicing region of intron 7 of the *LAMA4* gene, and one variant on the first nucleotide of the 3′-UTR of the *RBM20* gene. It is noteworthy that the most frequently mutated genes found in patients with HCM were *TTN*, *MYH7*, and *RBM20* (Figure 2A). Figure 2B shows the results of variants found per gene in the seven families with DCM. A total of fourteen DNA variants were found in 11 genes, where eleven were missense variants, two were frameshift variants in the *TTN* and *LMNA* genes, and one was an in frame coding indel in the *NEXN* gene. In this case, *TTN* and *MYH7* were the most frequently mutated genes. In the three families with ARVC, we found three missense variants, while the same frameshift variant was found in the *DSC2* gene in two unrelated families (Figure 2C). In the non-compaction cardiomyopathy family, one missense variant was found in the *TNNC1* gene (Figure 2D).

The 41 different non-synonymous variants are described in Table 2, which also includes the carrier status of family members. The missense variant c.4985G>A, p.Arg1662His in the *MYH7* gene was found in two families, one with HCM and one with DCM. Additionally, in two families with ARVC, we found the same frameshift variant, c.133delG, p.Ala45ProfsTer10, in the *DSC2* gene. It was interesting that in another family, FAM16 diagnosed with DCM, the missense variant found (*LAMP2*:c.3G>C, p.Met1Ile) was carried only by the tested proband and not by other family members nor by the parents. There was no evidence of non-paternity. Therefore, it could be a de novo variant. Moreover, it is noted that 15 out of 25 probands carried multiple variants (2–3) in multiple genes. Although we placed much weight on family segregation data, not all pedigrees provided unequivocal information regarding the concordance of the phenotype with the candidate pathogenic variants found in the proband’s DNA sample.

Table 3 shows the analysis of each validated variant with various tools, including global population databases. Importantly, each variant frequency was calculated against a sample of 1100 subjects of the general Cypriot population. It was interesting that some variants present in the Cypriot population are very rare in the global database of gnomAD Exomes. Finally, we classified each variant as a “variant of uncertain significance (VUS)”, “likely pathogenic” or “pathogenic”, per the ACMG criteria [36]. “Our classification” was based on various parameters, such as family segregation, the frequency in the global and in-house population databases, and the classification by Franklin and ClinVar (Table 3 and Table S2). The results for the forty-one different variants were as follows: twenty-eight (68.3%) variants were characterized as VUS, eight (19.5%) as likely pathogenic, and five (12.2%) as pathogenic. 

In 15 of the 25 families described here, there was at least one DNA variant that was previously reported in the HGMD professional database as (DM) or (DM?) in cardiac diseases. More specifically, 12 different variants were characterized as (DM), whereas three of them as (DM?). Although most of the (DM) and (DM?) variants were most probably disease-causing variants, in certain cases, there were conflicting reports regarding their pathogenicity by more than one author, especially with the (DM?) variants. Therefore, we classified the twelve different (DM) variants as follows: five variants as VUS, three as likely pathogenic, and four as pathogenic. On the other hand, two out of three (DM?) variants were classified as VUS due to the lack of a sufficient interpretation of the variant data and only one was classified as likely pathogenic (Table 3).

Interestingly, of all variants found, 26 were never reported in patients with cardiac diseases archived in the HGMD professional database, thus expanding the mutational spectrum of genes linked to inherited cardiomyopathies. Specifically, 10 of them were novel, currently absent in any public databases, whereas the remaining 16 were previously reported in the dbSNP or gnomAD Exomes general population databases, most of them with low or very low frequency. Regarding the ten novel variants, five of them were classified as VUS, four as likely pathogenic, and one as pathogenic. The 16 variants previously reported in population databases were all characterized as VUS (Table 3).

### 3.3. Family Studies

We describe analytically the variants found in three families with a cardiac phenotype and how they were inherited among the family members alongside their clinical symptoms.

**Figure 3:**
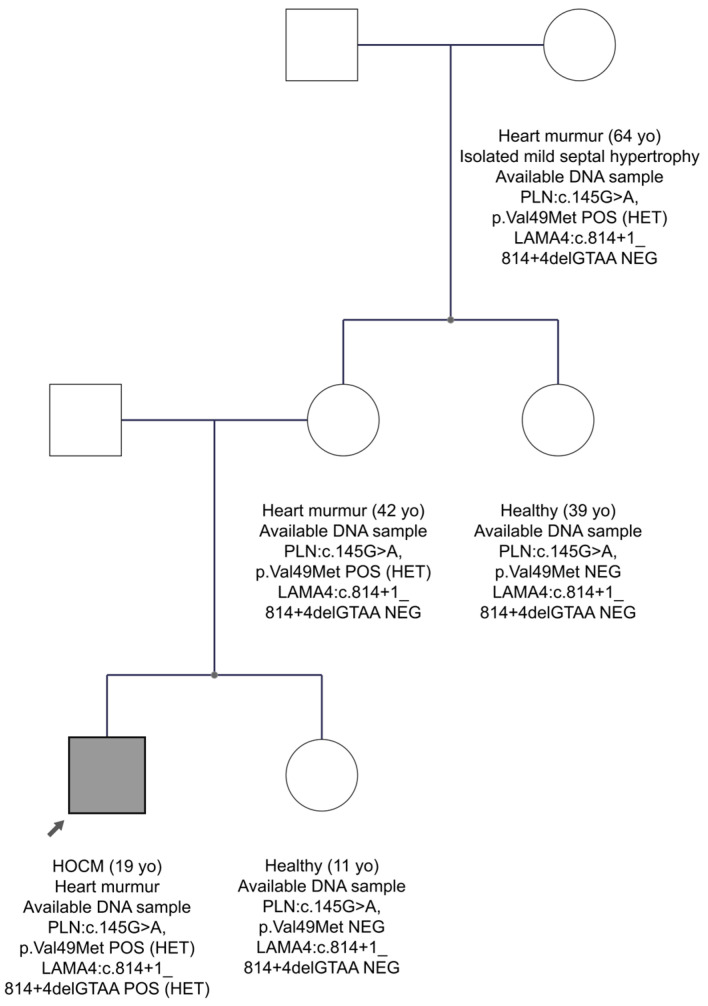
The pedigree of FAM10. The proband is indicated by the black arrow. Information provided below each subject is the clinical data, age, the gene, and variant(s) found. Circles are females and squares are males. Filled symbols are clinically affected subjects.

In family FAM10 with the HCM diagnosis, we found two candidate variants, one in the *LAMA4* gene and another one in the *PLN* gene, in the proband’s sample. The *LAMA4* gene, which encodes the laminin α4 chain, is an essential component of the extracellular matrix laminin-8 and -9 and has structural and signalling roles [37]. However, mutations in the *LAMA4* gene have been identified in patients with DCM [38]. The *PLN* gene encodes phospholamban that regulates the sarcoplasmic reticulum Ca^2+^-ATPase (SERCA), which transports calcium from the cytosol into the sarcoplasmic reticulum [39]. Mutations in the *PLN* gene are implicated in HCM [40]. The *LAMA4* variant was a four-nucleotide deletion encompassing the consensus donor splice sequence in intron 7 (*LAMA4*:c.814+1_814+4delGTAA). This variant is very rare in the gnomAD Exomes database, as well as in the Cypriot population, 0.0009 (1/1111). The professional HGMD database does not report the variant, whereas Franklin characterizes it as VUS to likely pathogenic. The second variant, *PLN*:c.145G>A, p.Val49Met (in exon 2), had a very low MAF score in the international population databases and it has not been detected in the Cypriot population. In addition, it is characterized as a disease-causing variant for HCM by the HGMD professional database. Specifically, the variant was first reported in a study by Xu J et al. and was found in one HCM patient [41]. Franklin characterizes it as VUS to likely pathogenic. The FAM10 family segregation is shown in Figure 3. The proband is the only person who has inherited the two variants in the *LAMA4* and *PLN* genes. At the age of 19, he was diagnosed with a heart murmur and hypertrophic obstructive cardiomyopathy (HOCM). On the other hand, his mother, aged 42, carries only the variant in the *PLN* gene and has a heart murmur. His grandmother at age 64 years, who also carries the variant in the *PLN* gene, has isolated mild septal hypertrophy and heart murmur. Hence, we suggest that the proband has a more severe phenotype than the other family members, perhaps due to the co-inheritance of the two variants. As the family data are not strong enough, the possibility remains that there is digenic inheritance, although not conclusively confirmed. Therefore, the *LAMA4* variant remains as a VUS waiting for stronger evidence.

**Figure 4:**
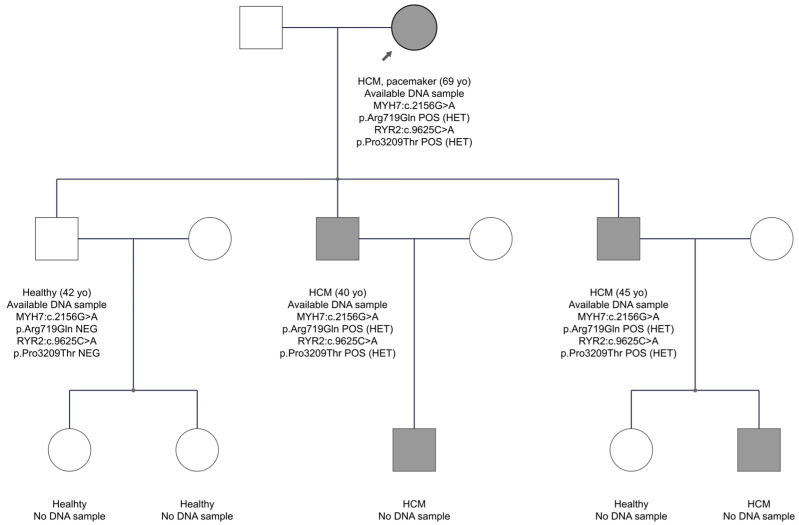
The pedigree of FAM11. The proband is indicated by the black arrow. Information provided below each subject is the clinical data, age, the gene, and variant(s) found. Note that three affected individuals inherited both variants, whereas the healthy brother inherited none.

In family FAM11 with the HCM diagnosis, two variants were identified in the *MYH7* and *RYR2* genes in the proband’s DNA. The *MYH7* gene is one of the two genes most commonly implicated in HCM. *RYR2* gene, which encodes the cardiac ryanodine receptor 2, a channel located on the sarcoplasmic reticulum that controls calcium release for myocardium contraction, is recently considered a causal gene for HCM [42]. The *MYH7* gene variant, exon 19, is c.2156G>A, p.Arg719Gln. It was not reported before in the gnomAD Exomes database, neither was it found in 1100 individuals of the Cypriot population. The HGMD database characterizes the variant as a disease-causing mutation for the development of hypertrophic cardiomyopathy. Several publications support this variant as pathogenic for HCM. In addition, the Franklin and ClinVar databases classify it as pathogenic. The second variant (c.9625C>A, p.Pro3209Thr) was in exon 68 of the *RYR2* gene, substituting proline with threonine at amino acid position 3209. The in-silico analysis shows that it is extremely rare in the gnomAD Exomes database, whereas the MAF score in the Cypriot general population is 0.004. Based on ACMG criteria, the Franklin database classifies it as VUS to likely pathogenic, and it has never been reported before in the HGMD professional database. Impressively, as shown in the pedigree of FAM11 (Figure 4), both variants co-segregate with the disease. Two sons of the proband, aged 40 and 45 years, have inherited both variants and have the disease, while a third son at age 42 years has inherited neither of the candidate variants and is healthy. These data support that the two variants may be responsible for the phenotype in this family.

Although *RYR2* variants are officially linked to ventricular arrhythmias and tachychardias (OMIM 115000 & OMIM 604772), in OMIM entry 180902, there is a reference to reports associating *RYR2* variants with catecholaminergic polymorphic ventricular tachycardia, sinoatrial and atrioventricular node dysfunction, atrial arrhythmias, and dilated cardiomyopathy, thus expanding the phenotypic spectrum of *RYR2*-related diseases. In FAM11, it is possible that we witness digenic inheritance, where trans-heterozygosity for two variants in two cardiac genes contributed to the phenotype, or the inheritance of two different genetic defects will manifest a mixed phenotype. At the same time, the phenotype could largely be explained by the single variant in the *MYH7* gene, and therefore the *RYR2* variant remains as a VUS waiting for additional evidence.

**Figure 5:**
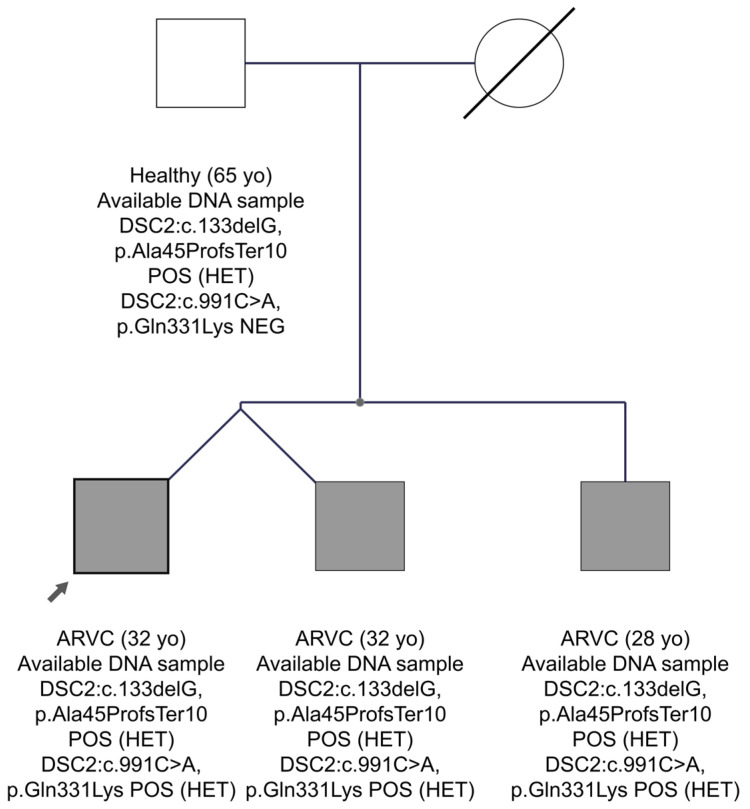
The pedigree of FAM24. The proband is indicated by the black arrow. Information provided below each subject is the clinical data, age, the gene, and variant(s) found.

In family FAM24 with the ARVC diagnosis, two candidate pathogenic variants were found in the *DSC2* gene in the proband’s sample. In general, the *DSC2* gene, which encodes the desmocollin-2 protein of the desmosomes, is frequently implicated in ARVC [43]. The first variant is a frameshift in exon 2, *DSC2*:c.133delG, p.Ala45ProfsTer10, which has not been reported before in the gnomAD Exomes database or in the Cypriot population. The HGMD database characterizes it as a disease-causing mutation for the development of ARVC. Franklin characterizes it as likely pathogenic and it has not been reported before in ClinVar. The second variant in the *DSC2* gene in exon 8 is c.991C>A, p.Gln331Lys. This variant has a MAF frequency in the Cypriot general population of 0.0004, whereas the international population databases do not report it. There is no report of the variant in both HGMD professional and ClinVar, whereas Franklin characterizes it as VUS. As shown in the pedigree of FAM24 (Figure 5), the proband aged 32 years, as well as his twin brother and younger brother aged 28 years, have inherited the two variants in the *DSC2* gene and have ARVC. His father at age 65 years carries only the frameshift variant and is healthy. Therefore, we concluded that the phenotype observed in the three brothers may be caused by the co-inheritance of the two variants in the *DSC2* gene, one from each parent, as an autosomal recessive condition. That the second variant, *DSC2*:c.991C>A, could have happened as a de novo event in the father’s sperm in cis to the first one and consequently he might be germinal mosaic remains as a very remote probability, considering the inheritance by three children.

## 4. Discussion

Inherited heart maladies represent an example of medical conditions characterized by genetic and allelic heterogeneity, a reality that makes genetic diagnosis highly challenging. In addition to the fact that disease-causing DNA variants in a plethora of genes can cause the same or a similar phenotype, a new emerging understanding is that Mendelian inheritance is now enriched with an ever-increasing suspicion of digenic or even oligogenic inheritance, as previously suggested [44]. The potential role of one primary gene, which is influenced by one or more co-inherited genetic modifiers, cannot be excluded. Additionally, owing to the high number of implicated genes, the co-inheritance of two or more variants and manifestation of a mixed phenotype is a likely scenario. Good large families with many affected subjects and deep phenotyping are needed to disentangle these complexities [45].

Variant evaluation: In this study, for the evaluation of each variant, we used the online Franklin and ClinVar tools (Table 3). Importantly, for variants previously published, we used information from the HGMD Professional database, which, although it is not always unequivocal, is an excellent source of information, especially when it contains clinical data of the patients who carry the respective variants. Based on all these, plus the family segregation and the frequencies in the European and Cypriot populations, we ended up with our suggested “private” classification (Table 3, last column). Publishing variants classified as VUS is a good practice, in our opinion, as it allows other researchers to cross-examine information and support or annul their potential pathogenic roles as more data accumulate.

We herewith attempted a systematic presentation of 25 families segregating inherited cardiomyopathies in Cyprus, for the first time accompanied with genetic investigations using the application of high-throughput massive parallel NGS analysis based on a panel of 72 genes. Apart from previous notable exceptions, no information is available about the spectrum of genetic lesions in Cypriot patients with inherited heart phenotypes. When we started this effort to fill a noticeable unmet need, we hypothesized that many (likely) pathogenic variants would be easily recognizable, based on the knowledge and the prior data accumulated over the previous decades in large laboratories around the world. In addition, we hypothesized that based on previous experience regarding Cypriot population genetics, we would discover one or more strong founder variants that would account for many families. Instead, we discovered that 68.3% of 41 different variants identified remain as VUS, which are equally probable to be or not to be pathogenic. Only two variants are shared by two unrelated families each; FAM23 and FAM24 with ARVC share variant *DSC2*:c.133delG, p.Ala45ProfsTer10; and FAM09 and FAM19, with HOCM and DCM, respectively, share variant *MYH7*:c.4985G>A, p.Arg1662His (Table 2). Importantly, the same variant in the *MYH7* gene was found in patients with HCM and DCM, a not unusual phenotypic diversity.

It is probable that the relatively small number of families tested thus far has not revealed yet wider founder mutations. This may change in the future. Examples of impressive founder pathogenic variants causing autosomal dominant conditions in the Cypriot population are the following: p.Gly1334Glu in the *COL4A3* gene (Alport syndrome and thin basement membrane nephropathy and focal segmental glomerulosclerosis) [46,47]; *CFHR5* exons 2–3 duplication (endemic complement C3 glomerulonephritis) [48]; a single C insertion amongst 7 cytosines in a complex sequence of a GC-rich variable number of tandem repeats of the *MUC1* gene (MUC1 kidney disease) [49,50]; and the p.Val30Met variant in the *TTR* gene, causing autosomal dominant sensorimotor and autonomic neuropathy because of amyloid deposition (ATTRV30M neuropathy) [51].

According to the literature, (likely) pathogenic variants in the *MYH7* and *MYBPC3* genes account for 35–65% of patients with HCM [52]. In the present study, variants in these genes account for 29% (4 of 14 families). Not surprisingly, owing to the length of the gene, being the largest gene with 363 exons, *TTN* had the most variants, seven in total in five families, found in both HCM and DCM patients. Five variants are missense mutations classified as VUS and one is a 3-nucleotide deletion also classified as VUS, all six being very rare or not found in the Cypriot population. The seventh variant in the *TTN* gene is a frameshift variant classified as likely pathogenic in a DCM patient. It was never reported (Figure 2 and Table 3).

Family segregation: Not all families were large enough to draw unequivocal conclusions regarding the concordance of phenotype with the genotype. It was not unusual to see variants inherited by subjects who remained healthy, even the parents of the proband, perhaps due to incomplete penetrance (see pedigrees FAM02, FAM04, FAM15, and FAM21—Figure S1). In several families, one of the parents of the proband had a recorded phenotype, including death, but no DNA was available for testing (see pedigrees FAM02 and FAM06—Figure S1). In cases of presumed digenic or oligogenic inheritance in the proband, children and other relatives might remain healthy because they did not inherit all (likely) pathogenic variants, or they only presented a partial phenotype (see pedigrees FAM15 and FAM20—Figure S1).

Other occasions where the children carrying the variant(s) remained healthy could be attributed to age-dependent penetrance (see pedigrees FAM02 and FAM21—Figure S1). Of course, one cannot preclude the likelihood that some recorded filtered variants classified as VUS are not pathogenic. At the same time, it is worth noting that for 15 of the 25 probands more than one variant was filtered in for our final verdict, suggesting either oligogenic inheritance or the co-inheritance of a primary variant with potential genetic modifiers that exacerbated or precipitated the phenotype. This is supported by the visual inspection of the pedigrees, where on multiple occasions, the healthy children or other siblings or first cousins who remain healthy have inherited only a subset of the variants that are co-inherited by the proband (see examples of pedigrees FAM09 and FAM15—Figure S1). 

## 5. Conclusions

A major challenge, which is a common experience by all experts, is incomplete penetrance and variable expressivity, while the multiplicity of variants found in a large repertoire of genes certainly complicates the offer of definite results in many cases. These are challenges that are easier to address when large families with subjects in multiple generations are available. Notwithstanding that in Cyprus, we still have easier access to large families and people are usually cooperative; our experience here has been somewhat disappointing as the inheritance of good candidate variants was not always met with unequivocal transgenerational transmission of the phenotype either because of age-dependent penetrance or reduced penetrance, oligogenic inheritance, or a combination of the above.

Importantly, the rare or very rare variants we reported here, which were previously reported in the HGMD database, could be of an ancestral relationship or the result of recurrence. We were not able to investigate this further. It is interesting though that the parallel massive genetic testing by groups around the world has led to the reporting of many variants, gradually leading to relative satiety. This, when accompanied by detailed clinical data, will gradually facilitate the interpretation of findings regarding their potential pathogenicity, thus re-classifying variants of uncertain significance (VUS) into benign or pathogenic. We and others decided to include in the final evaluation of variants “Our Classification” (see Table 3 and Table S2), which is subject to confirmation or rejection based on new data to emerge from our or others’ work.

It is equally important to observe that considering the limited knowledge on the genetics of inherited heart conditions in Cyprus, the close follow-up of these and more patients to be tested genetically in our setting will enable better evaluations and the classification of DNA variants and their likely roles in disease phenotypes, thus contributing to precision cardiology. Finally, the availability of data from the Cypriot whole exome sequencing project (CYPROME) empowers the evaluation of the identified variants regarding the frequency of the minor allele in the very population under study and their potential associations with a phenotype through data mining of the Cyprus Biobank. One also hopes that the development of robust functional studies in cell cultures and/or animal models will further facilitate the interpretation of the variants.

## Figures and Tables

**Table 1:** The NGS panel.

The 72-Gene Panel								
*ABCC9*	*CACNA1C*	*DSC2*	*GLA*	*KCNQ1*	*MYL2*	*NKX2.5*	*RBM20*	*TMPO*
*ACTA1*	*CACNB2*	*DSG2*	*HCN4*	*LAMA4*	*MYL3*	*PDLIM3*	*RYR2*	*TNNC1*
*ACTC1*	*CASQ2*	*DSP*	*JPH2*	*LAMP2*	*MYLK2*	*PKP2*	*SCN5A*	*TNNI3*
*ACTN2*	*CAV3*	*DTNA*	*JUP*	*LDB3*	*MYOM1*	*PLN*	*SGCD*	*TNNT2*
*ALPK3*	*CRYAB*	*EMD*	*KCNE1*	*LMNA*	*MYOZ2*	*PRDM16*	*TAZ*	*TPM1*
*ANK2*	*CSRP3*	*EYA4*	*KCNE2*	*MYBPC3*	*MYPN*	*PRKAG2*	*TCAP*	*TTN*
*ANKRD1*	*DES*	*FKTN*	*KCNH2*	*MYH6*	*NEBL*	*PTPN11*	*TGFB3*	*TTR*
*BAG3*	*DMD*	*FLNC*	*KCNJ2*	*MYH7*	*NEXN*	*RAF1*	*TMEM43*	*VCL*

**Table 2:** Families with clinical phenotypes, demographic data, and genetic variants found in each patient proband. Family members include only at-risk members; married-in subjects are excluded. The question mark for the patients in FAM13, mean that 3 subjects had ambiguous phenotype.

Family Number	Clinical Phenotype	Number of Family Members with Available DNA Samples			Gender of the Proband	Age at Diagnosis of the Proband	Genetic Variants Found in the Proband	Number of Family Members with the Genetic Variants		
		Total	Patients	Healthy				Total	Clinically Affected Carriers	Clinically Healthy Carriers
**FAM01**	HCM	1 (1 M/0 F)	1 (1 M/0 F)	0	M	60	*ALPK3*:c.5548A>G, p.Lys1850Glu (HET)	1 (1 M/0 F)	1 (1 M/0 F)	0
**FAM02**	HCM	9 (5 M/4 F)	1 (1 M/0 F)	8 (4 M/4 F)	M	64	*VCL*:c.2415_2421delTGGAAAC, p.Gly806PhefsTer47 (HET)	4 (2 M/2 F)	1 (1 M/0 F)	3 (1 M/2 F)
**FAM03**	HOCM	2 (2 M/0 F)	1 (1 M/0 F)	1 (1 M/0 F)	M	63	*TNNI3*:c.428C>A, p.Thr143Asn (HET)	1 (1 M/0 F)	1 (1 M/0 F)	0
							*TTN*:c.81809_81811delAAG, p.Glu27270del (HET)	2 (2 M/0 F)	1 (1 M/0 F)	1 (1 M/0 F)
**FAM04**	HCM	10 (5 M/5 F)	2 (0 M/2 F)	8 (5 M/3 F)	F	62	*FLNC*:c.2635C>T, p.Arg879Cys (HET)	4 (1 M/3 F)	1 (0 M/1 F)	3 (1 M/2 F)
							*MYPN*:c.3959T>C, p.Leu1320Pro (HET)	2 (0 M/2 F)	1 (0 M/1 F)	1 (0 M/1 F)
**FAM05**	HCM	2 (1 M/1 F)	1 (1 M/0 F)	1 (0 M/1 F)	M	15	*MYPBC3*:c.3697C>T, p.Gln1233Ter (HET)	1 (1 M/0 F)	1 (1 M/0 F)	0
**FAM06**	HCM	9 (6 M/3 F)	1 (1 M/0 F)	8 (5 M/3 F)	M	10	*MYH7*:c.1357C>A, p.Arg453Ser (HET)	1 (1 M/0 F)	1 (1 M/0 F)	0
							*RBM20*:c.3584C>A, p.Ser1195Tyr (HET)	1 (1 M/0 F)	1 (1 M/0 F)	0
**FAM07**	HCM	2 (2 M/0 F)	1 (1 M/0 F)	1 (1 M/0 F)	M	50	*RBM20*:c.2761A>G, p.Ile921Val (HET)	1 (1 M/0 F)	1 (1 M/0 F)	0
**FAM08**	HOCM	3 (1 M/2 F)	2 (0 M/2 F)	1 (1 M/0 F)	F	65	*RBM20*:c.*1T>G (in the 3-UTR) (HET) *	2 (0 M/2 F)	2 (0 M/2 F)	0
**FAM09**	HOCM	6 (3 M/3 F)	1 (0 M/1 F)	5 (3 M/2 F)	F	60	*KCNQ1*:c.1768G>A, p.Ala590Thr (HET)	2 (1 M/1 F)	1 (0 M/1 F)	1 (1 M/0 F)
							*MYH7*:c.4985G>A, p.Arg1662His (HET)	4 (1 M/3 F)	1 (0 M/1 F)	3 (1 M/2 F)
							*DSC2*:c.1891A>G, p.Thr631Ala (HET)	5 (2 M/3 F)	1 (0 M/1 F)	4 (2 M/2 F)
**FAM10**	HOCM	5 (1 M/4 F)	1 (1 M/0 F)	4 (0 M/4 F)	M	13	*PLN*:c.145G>A, p.Val49Met (HET)	3 (1 M/2 F)	1 (1 M/0 F)	2 (0 M/2 F)
							*LAMA4*:c.814+1_814+4delGTAA (HET)	1 (1 M/0 F)	1 (1 M/0 F)	0
**FAM11**	HCM	4 (3 M/1 F)	3 (2 M/1 F)	1 (1 M/0 F)	F	35	*MYH7*:2156G>A, p.Arg719Gln (HET)	3 (2 M/1 F)	3 (2 M/1 F)	0
							*RYR2*:c.9625C>A, p.Pro3209Thr (HET)	3 (2 M/1 F)	3 (2 M/1 F)	0
**FAM12**	HCM	2 (1 M/1 F)	2 (1 M/1 F)	0	M	35	*TTN*:c.53273G>C, p.Arg17758Pro (HET)	1 (1 M/0 F)	1 (1 M/0 F)	0
							*TTN*:c.35271G>C, p.Glu11757Asp (HET)	2 (1 M/1 F)	2 (1 M/1 F)	0
**FAM13**	HCM	5 (3 M/2 F)	1, 3? (2 M/2 F)	1 (1 M/0 F)	M	35	*ALPK3*:c.4094C>T, p.Ala1365Val (HET)	3 (2 M/1 F)	1, 2? (2 M/1 F)	0
**FAM14**	HCM	3 (2 M/1 F)	1 (1 M/0 F)	2 (1 M/1 F)	M	19	*TTN*:c.22718G>T, p.Arg7573Ile (HET)	2 (2 M/0 F)	1 (1 M/0 F)	1 (1 M/0 F)
							*DSP*:c.7154G>A, p.Arg2385His (HET)	2 (1 M/1 F)	1 (1 M/0 F)	1 (0 M/1 F)
**FAM15**	DCM	6 (2 M/4 F)	1 (0 M/1 F)	5 (2 M/3 F)	F	65	*ANK2*:c.11458C>T, p.Arg3820Trp (HET)	3 (0 M/3 F)	1 (0 M/1 F)	2 (0 M/2 F)
							*DSP*:c.5324G>T, p.Arg1775Ile (HET)	3 (1 M/2 F)	1 (0 M/1 F)	2 (1 M/1 F)
**FAM16**	DCM	6 (1 M/5 F)	1 (0 M/1 F)	5 (1 M/4 F)	F	28	*LAMP2*:c.3G>C, p.Met1Ile (HET)	1 (0 M/1 F)	1 (0 M/1 F)	0
**FAM17**	DCM	7 (5 M/2 F)	3 (2 M/1 F)	4 (3 M/1 F)	F	38	*NEBL*:c.2513T>C, p.Ile838Thr (HET)	4 (2 M/2 F)	2 (1 M/1 F)	2 (1 M/1 F)
							*MYL2*:c.359G>A, p.Arg120Gln (HET)	3 (2 M/1 F)	2 (1 M/1 F)	1 (1 M/0 F)
**FAM18**	DCM	2 (2 M/0 F)	1 (1 M/0 F)	1 (1 M/0 F)	M	53	*LMNA*:c.908_909delCT, p.Ser303CysfsTer (HET)	1 (1 M/0 F)	1 (1 M/0 F)	0
**FAM19**	DCM	3 (3 M/0 F)	1 (1 M/0 F)	2 (2 M/0 F)	M	19	*MYH7*:c.2290T>C, p.Phe764Leu (HET)	1 (1 M/0 F)	1 (1 M/0 F)	0
							*MYH7*:c.4985G>A, p.Arg1662His (HET)	1 (1 M/0 F)	1 (1 M/0 F)	0
							*SCN5A*:c.5086C>T, p.Leu1696Phe (HET)	2 (2 M/0 F)	1 (1 M/0 F)	1 (1 M/0 F)
**FAM20**	DCM	3 (1 M/2 F)	1 (1 M/0 F)	2 (0 M/2 F)	M	65	*NEXN*:c.1582_1584delGAA, p.Glu528del (HET)	2 (1 M/1 F)	1 (1 M/0 F)	1 (0 M/1 F)
							*TTN*:c.51560A>C, p.Asn17187Thr (HET)	2 (1 M/1 F)	1 (1 M/0 F)	1 (0 M/1 F)
							*TTN*:c.88802G>A, p.Arg29601His (HET)	2 (1 M/1 F)	1 (1 M/0 F)	1 (0 M/1 F)
**FAM21**	DCM	8 (5 M/3 F)	1 (0 M/1 F)	7 (5 M/2 F)	F	70	*TTN*:c.87043_87044insCA, p.Ile29015ThrfsTer15 (HET)	5 (4 M/1 F)	1 (0 M/1 F)	4 (4 M/0 F)
							*PKP2*:c.184C>A, p.Gln62Lys (HET)	5 (3 M/2 F)	1 (0 M/1 F)	4 (3 M/1 F)
**FAM22**	ARVC	1 (1 M/0 F)	1 (1 M/0 F)	0	M	40	*DES*:c.128A>C, p.Lys43Thr (HET)	1 (1 M/0 F)	1 (1 M/0 F)	0
**FAM23**	ARVC	4 (2 M/2 F)	0	4 (2 M/2 F)	F	The proband is healthy but she has a positive family history of ARVC	*DSC2*:c.133delG, p.Ala45ProfsTer10 (HET)	2 (1 M/1 F)	0	2 (1 M/1 F)
							*MYH6*:c.5072G>C, p.Arg1691Pro (HET)	3 (1 M/2 F)	0	3 (1 M/2 F)
**FAM24**	ARVC	4 (4 M/0 F)	3 (3 M/0 F)	1 (1 M/0 F)	M	20	*DSC2*:c.133delG, p.Ala45ProfsTer10 (HET)	4 (4 M/0 F)	3 (3 M/0 F)	1 (1 M/0 F)
							*DSC2*:c.991C>A, p.Gln331Lys (HET)	3 (3 M/0 F)	3 (3 M/0 F)	0 (0 M/0 F)
**FAM25**	NCM	5 (3 M/2 F)	1 (1 M/0 F)	4 (2 M/2 F)	M	19	*TNNC1*:c.435C>A, p.Asp145Glu (HET)	2 (2 M/0 F)	1 (1 M/0 F)	1 (1 M/0 F)

* The variant *RBM20*:c*1T>G in FAM08 was excluded from the filtering steps but included in the analysis because it is a novel finding and is characterized as VUS in the Franklin tool. HOCM: hypertrophic obstructive cardiomyopathy, M: male, F: female. ?: The question mark for the patients in FAM13, means that three subjects had ambiguous phenotype.

**Table 3:** Details about DNA variants found in each family and evaluations with several online tools. Included are the Minor Allele Frequency (MAF) in global databases, as well as the MAF in the Cypriot population (CY-MAF, CYPROME). In the final column, Our Classification is based on the classification by Franklin and ClinVar, adopting the worst scenario in combination with the variant population frequency and our family segregation data (see also Table S2). In the text, the “HGMD Professional” finding and citation are also discussed, if available.

Family Number	Gene (Exon)	Chromosome Position; Transcript	Coding	Protein	dbSNP Entry	MAF (1000 Genomes)	MAF (GnomAD Exomes)	CY-MAF (Based on 1100 Samples)	HGMD Professional	Franklin	ClinVar	Our Classification
**FAM01** (HCM)	*ALPK3* (Exon 14)	chr15:85411511; NM_020778.4	c.5548A>G	p.Lys1850Glu	rs1273857977	Not reported	Not reported	Not found	Not reported	VUS to likely benign	Not reported	VUS
**FAM02** (HCM)	*VCL* (Exon 16)	chr10:75865090; NM_014000.2	c.2415_2421delTGGAAAC	p.Gly806PhefsTer47	Not reported	Not reported	Not reported	0.000454545	Not reported	Likely pathogenic	Not reported	Likely pathogenic
**FAM03** (HOCM)	*TNNI3* (Exon 7)	chr19:55665519; NM_000363.4	c.428C>A	p.Thr143Asn	rs397516348	Not reported	0.0000362	Not found	DM? cardiomyopathy, hypertrophic CM135615	VUS to likely pathogenic	Conflicting interpretations of pathogenicity	Likely pathogenic
	*TTN* (Exon 276)	chr2:179424125; NM_001256850.1	c.81809_81811delAAG	p.Glu27270del	rs727504797	Not reported	0.000169	Not found	Not reported	VUS to likely pathogenic	Conflicting interpretations of pathogenicity	VUS
**FAM04** (HCM)	*FLNC* (Exon 17)	chr7:128483367; NM_001458.4	c.2635C>T	p.Arg879Cys	rs374983276	Not reported	0.000122	0.000454545	Not reported	VUS	Conflicting interpretations of pathogenicity	VUS
	*MYPN* (Exon 20)	chr10:69970208; NM_001256267.1	c.3959T>C	p.Leu1320Pro	rs200646285	0.0002	0.00000796	0.001818182	DM? noncompaction, left ventricular CM1711700	VUS	Uncertain significance	VUS
**FAM05** (HCM)	*MYBPC3* (Exon 33)	chr11:47353740; NM_000256.3	c.3697C>T	p.Gln1233Ter	rs397516037	Not reported	0.00000802	Not found	DM cardiomyopathy, hypertrophic CM014069	Pathogenic	Pathogenic/ Likely pathogenic	Pathogenic
**FAM06** (HCM)	*MYH7* (Exon 14)	chr14:23898214; NM_000257.3	c.1357C>A	p.Arg453Ser	rs121913625	Not reported	Not reported	Not found	DM cardiomyopathy, hypertrophic CM087715	Pathogenic	Pathogenic	Pathogenic
	*RBM20* (Exon 14)	chr10:112595636; NM_001134363.2	c.3584C>A	p.Ser1195Tyr	rs753102653	Not reported	0.000278	0.002727273	DM? noncompaction, left ventricular CM1711693	VUS to likely benign	Conflicting interpretations of pathogenicity	VUS
**FAM07** (HCM)	*RBM20* (Exon 11)	chr10:112581138; NM_001134363.2	c.2761A>G	p.Ile921Val	rs397516608	0.000399	0.0000702	0.000454545	Not reported but DM? cardiomyopathy, non-compaction with c.2761A>T, p.Ile921Phe CM1924052	VUS	Conflicting interpretations of pathogenicity	VUS
**FAM08** (HOCM)	*RBM20*	chr10:112595737; NM_001134363.2	c.*1T>G (in the 3′-UTR)	/	Not reported	Not reported	Not reported	Not found	Not reported	VUS to likely benign	Not reported	VUS
**FAM09** (HOCM)	*KCNQ1* (Exon 15)	chr11:2799241; NM_000218.2	c.1768G>A	p.Ala590Thr	rs199472813	Not reported	0.00000797	0.000454545	DM long QT syndrome CM040442	Pathogenic	Conflicting interpretations of pathogenicity	Likely pathogenic
	*MYH7* (Exon 35)	chr14:23885010; NM_000257.3	c.4985G>A	p.Arg1662His	rs370328209	Not reported	0.0000597	0.000454545	DM cardiomyopathy, dilated CM115875	VUS to likely pathogenic	Conflicting interpretations of pathogenicity	VUS
	*DSC2* (Exon 13)	chr18:28651805; NM_024422.4	c.1891A>G	p.Thr631Ala	Not reported	Not reported	Not reported	0.004090909	Not reported	VUS	Not reported	VUS
**FAM10** (HOCM)	*PLN* (Exon 2)	chr6:118880229; NM_002667.4	c.145G>A	p.Val49Met	rs749962743	Not reported	0.0000119	Not found	DM cardiomyopathy, hypertrophic CM1513486	VUS to likely pathogenic	Uncertain significance	Likely pathogenic
	*LAMA4*	chr6:112510308; NM_001105206.2	c.814+1_814+4delGTAA	/	rs782628388	Not reported	0.00002124	0.000909091	Not reported	VUS to likely pathogenic	Not reported	VUS
**FAM11** (HCM)	*MYH7* (Exon 19)	chr14:23895179; NM_000257.3	c.2156G>A	p.Arg719Gln	rs121913641	Not reported	Not reported	Not found	DM cardiomyopathy, hypertrophic CM941085	Pathogenic	Pathogenic	Pathogenic
	*RYR2* (Exon 68)	chr1:237870293; NM_001035.2	c.9625C>A	p.Pro3209Thr	rs767375014	Not reported	0.00000803	0.004090909	Not reported but DM? sudden infant death syndrome with c.9626C>T, p.Pro3209Leu CM1824622	VUS to likely pathogenic	Uncertain significance	VUS
**FAM12** (HCM)	*TTN* (Exon 247)	chr2:179458924; NM_001256850.1	c.53273G>C	p.Arg17758Pro	Not reported	Not reported	Not reported	0.001363636	Not reported but DM? cardiomyopathy with c.53273G>A, p.Arg17758Gln CM1924179	VUS	Not reported	VUS
	*TTN* (Exon 165)	chr2:179514916; NM_001256850.1	c.35271G>C	p.Glu11757Asp	rs1442749271	Not reported	0.00000465	Not found	Not reported	VUS	Not reported	VUS
**FAM13** (HCM)	*ALPK3* (Exon 6)	chr15:85401457; NM_020778.4	c.4094C>T	p.Ala1365Val	rs755941827	Not reported	0.0000496	0.001818182	Not reported	VUS to likely benign	Uncertain significance	VUS
**FAM14** (HCM)	*TTN* (Exon 80)	chr2:179584550; NM_001256850.1	c.22718G>T	p.Arg7573Ile	rs370939248	Not reported	0.0000124	Not found	Not reported	VUS to likely pathogenic	Uncertain significance	VUS
	*DSP* (Exon 24)	chr6:7584649; NM_004415.3	c.7154G>A	p.Arg2385His	rs1396768987	Not reported	0.00000398	Not found	Not reported	VUS to likely benign	Likely benign	VUS
**FAM15** (DCM)	*ANK2* (Exon 43)	chr4:114290809; NM_001148.5	c.11458C>T	p.Arg3820Trp	rs199922285	0.000399	0.0000239	0.009545455	Not reported	VUS	Not reported	VUS
	*DSP* (Exon 23)	chr6:7581747; NM_004415.3	c.5324G>T	p.Arg1775Ile	rs34738426	0.0002	0.0000678	0.007727273	DM cardiomyopathy, arrhythmogenic right ventricular CM056324	VUS to likely benign	Conflicting interpretations of pathogenicity	VUS
**FAM16** (DCM)	*LAMP2* (Exon 1)	chrX:119603022; NM_001122606.1	c.3G>C	p.Met1Ile	Not reported	Not reported	Not reported	Not found	Not reported	Likely pathogenic	Not reported	Likely pathogenic
**FAM17** (DCM)	*NEBL* (Exon 24)	chr10:21101703; NM_006393.2	c.2513T>C	p.Ile838Thr	rs749452317	Not reported	0.00000398	Not found	Not reported	VUS	Uncertain significance	VUS
	*MYL2* (Exon 6)	chr12:111350943; NM_000432.3	c.359G>A	p.Arg120Gln	rs192057022	0.000399	0.0000517	0.000454545	Not reported but DM cardiomyopathy, hypertrophic with c.358C>T, p.Arg120Trp CM1617083	VUS to likely benign	Conflicting interpretations of pathogenicity	VUS
**FAM18** (DCM)	*LMNA (Exon 5)*	chr1:156105070; NM_170707.3	c.908_909delCT	p.Ser303CysfsTer27	rs59684335	Not reported	Not reported	Not found	DM cardiomyopathy, dilated CD035724	Pathogenic	Pathogenic	Pathogenic
**FAM19** (DCM)	*MYH7* (Exon 21)	chr14:23894624; NM_000257.3	c.2290T>C	p.Phe764Leu	Not reported	Not reported	Not reported	Not found	Not reported but DM cardiomyopathy with c.2292C>A, p.Phe764Leu CM2037625 & DM cardiomyopathy, dilated with c.2292C>G, p.Phe764Leu CM003003 & cardiomyopathy, hypertrophic with c.2291T>A, p.Phe764Tyr CM1310641	Pathogenic	Not reported	Pathogenic
	*MYH7* (Exon 35)	chr14:23885010; NM_000257.3	c.4985G>A	p.Arg1662His	rs370328209	Not reported	0.0000597	0.000454545	DM cardiomyopathy, dilated CM115875	VUS to likely pathogenic	Conflicting interpretations of pathogenicity	VUS
	*SCN5A* (Exon 27)	chr3:38592615; NM_001160161.1	c.5086C>T	p.Leu1696Phe	rs45606037	Not reported	0.00000398	Not found	Not reported	VUS to likely pathogenic	Not reported	VUS
**FAM20** (DCM)	*NEXN* (Exon 12)	chr1:78407806; NM_144573.3	c.1582_1584delGAA	p.Glu528del	rs764505909	Not reported	0.000153	Not found	DM cardiomyopathy dilated CD1315240	VUS to likely pathogenic	Uncertain significance	VUS
	*TTN* (Exon 240)	chr2:179464037; NM_001256850.1	c.51560A>C	p.Asn17187Thr	rs71423569	Not reported	Not reported	0.000909091	Not reported	VUS	Not reported	VUS
	*TTN* (Exon 289)	chr2:179412628; NM_001256850.1	c.88802G>A	p.Arg29601His	rs369899675	Not reported	0.000101	Not found	Not reported	VUS to likely benign	Conflicting interpretations of pathogenicity	VUS
**FAM21** (DCM)	*TTN* (Exon 288)	chr2:179414482; NM_001256850.1	c.87043_87044insCA	p.Ile29015ThrfsTer15	Not reported	Not reported	Not reported	Not found	Not reported	Likely pathogenic	Not reported	Likely pathogenic
	*PKP2* (Exon 1)	chr12:33049482; NM_004572.3	c.184C>A	p.Gln62Lys	rs199601548	Not reported	0.000141	Not found	DM arrhythmogenic right ventricular dysplasia CM061171	VUS	Conflicting interpretations of pathogenicity	VUS
**FAM22** (ARVC)	*DES* (Exon 1)	chr2:220283312; NM_001927.3	c.128A>C	p.Lys43Thr	Not reported	Not reported	Not reported	Not found	Not reported but DM? cardiomyopathy, dilated with c.127A>G, p.Lys43Glu CM1616845	VUS to likely pathogenic	Not reported	VUS
**FAM23** (ARVC)	*DSC2* (Exon 2)	chr18:28673542; NM_024422.4	c.133delG	p.Ala45ProfsTer10	rs1460932284	Not reported	Not reported	Not found	DM arrhythmogenic right ventricular cardiomyopathy CD1925890	Likely pathogenic	Not reported	Likely pathogenic
	*MYH6* (Exon 34)	chr14:23855228; NM_002471.3	c.5072G>C	p.Arg1691Pro	Not reported	Not reported	Not reported	0.000909091	Not reported but DM cardiomyopathy, hypertrophic with c.5071C>T, p.Arg1691Cys CM1813213 & DM? cardiomyopathy, hypertrophic with c.5072G>A, p.Arg1691His CM204938	VUS to likely pathogenic	Not reported	VUS
**FAM24** (ARVC)	*DSC2* (Exon 2)	chr18:28673542; NM_024422.4	c.133delG	p.Ala45ProfsTer10	rs1460932284	Not reported	Not reported	Not found	DM arrhythmogenic right ventricular cardiomyopathy CD1925890	Likely pathogenic	Not reported	Likely pathogenic
	*DSC2* (Exon 8)	chr18:28662978; NM_024422.4	c.991C>A	p.Gln331Lys	Not reported	Not reported	Not reported	0.000454545	Not reported	VUS	Not reported	Likely pathogenic
**FAM25** (Non-compaction cardiomyopathy)	*TNNC1* (Exon 5)	chr3:52485426; NM_003280.2	c.435C>A	p.Asp145Glu	rs267607124	Not reported	0.000132	Not found	DM cardiomyopathy, hypertrophic CM083569	Likely pathogenic	Uncertain significance	VUS

HOCM: hypertrophic obstructive cardiomyopathy.

## Data Availability

Data concerning the results published here are available by contacting the corresponding authors. The data are not publicly available due to privacy and ethical reasons.

## Supplementary Materials

The following supporting information can be downloaded at https://www.mdpi.com/article/10.3390/genes15030319/s1: Table S1. The 72 genes in our NGS panel and their associated general and clinical features; Table S2. Variants that, based on our data, were upgraded to likely pathogenic and a description of the relevant ACMG criteria; Figure S1. Pedigrees of FAM01-25 described in the paper.
