# Supplementary material for: Massive Parallel DNA Sequencing of Patients with Inherited Cardiomyopathies in Cyprus and Suggestion of Digenic or Oligogenic Inheritance
Source: Genes (Basel). 2024 Feb 28;15(3):319. doi: 10.3390/genes15030319 (PMC10970479; doi:10.3390/genes15030319)
Supplement: Supplementary file 1 [file genes-15-00319-s001.zip › genes-2711172-supplementary.pdf]

Supplemental Materials

Table S1. The 72 genes in our NGS panel and their associated general and clinical features

Gene	Protein	Role of protein in cardiomyocytes	Inheritance	HCM	DCM	RCM	ARVC	LVNC
ABCC9	Sulfonylurea receptor 2 (SUR2)	Involving in the formation of potassium channels	AD		X			
ACTA1	Actin alpha 1, skeletal muscle	The core of the thin filament of the sarcomere in adult skeletal muscle	AD, AR	X				
ACTC1	Actin alpha 1, cardiac muscle	The core of the thin filament of the sarcomere in adult cardiac muscle	AD	X	X	X		X
ACTN2	Actinin alpha 2	Structural and functional role in the sarcomeres	AD	X	X			
ALPK3	Alpha protein kinase 3	Transcriptional regulator which phosphorylates cardiac transcriptional factors; involving in cardiac muscle cell development	AR	X				
ANK2	Ankyrin B	Interacting with ion channels, ion transporters and signalling molecules across the cell membranes	AD					
ANKRD1	Ankyrin repeat protein	Z-disc component	N/A	X	X			
BAG3	BCL2 associated athanogene 3	Co-chaperone with members of the heat-shock protein family; inhibiting apoptosis; Z-disc structural role	AD	X	X	X		
CACNA1C	Calcium voltage-gated channel subunit alpha 1	Involving in the formation of calcium channels	AD					
CACNB2	Calcium voltage-gated channel subunit beta 2	Involving in the formation of calcium channels	AD					

CASQ2	Calsequestrin 2	Involving in the storage and transport of calcium ions through the sarcoplasmic reticulum	AR					X
CAV3	Caveolin 3	Component of the plasma membrane of muscle cells; regulating ion channels; playing role in signal transduction	AD, DD	X	X			
CRYAB	Crystallin alpha B	Acting as a molecular chaperone	AD, AR	X	X			
CSRP3	Cysteine and glycine rich protein 3	Z-disc structural role; implicating in transcriptional regulation, cytoskeleton organization, cell adhesion and signal transduction	AD	X	X			
DES	Desmin	Structural and mechanical integrity of the contractile apparatus	AD, AR		X	X	X	
DMD	Dystrophin	Part of the dystrophin-glycoprotein complex, which bridges the cytoskeleton and the extracellular matrix	XLR, XL		X			X
DSC2	Desmocollin 2	Component of the desmosome, which provides adhesion between cardiac cells	AD, AR		X		X	
DSG2	Desmoglein 2	Component of the desmosome, which provides adhesion between cardiac cells	AD		X		X	
DSP	Desmoplakin	Linking the desmosomes to intermediate filaments	AD, AR		X		X	
DTNA	Dystrobrevin alpha	Part of the dystrophin-glycoprotein complex, which bridges the cytoskeleton and the extracellular matrix	AD					X
EMD	Emerin	Component of the nuclear envelope	XLR		X			
EYA4	EYA transcriptional coactivator and phosphatase 4	Acting as a transcriptional activator	AD		X			

FKTN	Fukutin	Ribitol 5-phosphate transferase in the cis-Golgi compartment involving in the glycosylation of alpha dystroglycan	AR		X			
FLNC	Filamin C	Crosslinking actin filaments and anchoring the plasma membrane proteins to the cytoskeleton	AD	X		X		
GLA	Galactosidase alpha	Hydrolysing the terminal alpha-galactose groups from glycoproteins and glycolipids in lysosomes	XL	X		X		X
HCN4	Hyperpolarization activated cyclic nucleotide gated potassium channel 4	Transporting ions into cells of the sino-atrial node, which are necessary for the generation of electrical impulses that start each heartbeat	AD					
JPH2	Junctophilin 2	Component of the junctional membrane complexes which connect the plasma membrane and the sarcoplasmic reticulum	AD	X				
JUP	Junction Plakoglobin	Component of the desmosome, which provides adhesion between cardiac cells	AD, AR				X	
KCNE1	Potassium voltage-gated channel subfamily E regulatory subunit 1	Binding to the potassium channels and regulating their activity	AD, AR					
KCNE2	Potassium voltage-gated channel subfamily E regulatory subunit 2	Binding to the potassium channels and regulating their activity	AD					
KCNH2	Potassium voltage-gated channel subfamily H member 2	Making up potassium channels	AD					
KCNJ2	Potassium voltage-gated channel subfamily J member 2	Making up potassium channels	AD					
KCNQ1	Potassium voltage-gated channel subfamily Q member 1	Making up potassium channels	AD					

LAMA4	Laminin subunit alpha 4	The major noncollagenous component of the basement membrane; implicating in cell adhesion, differentiation, migration, signalling and metastasis	AD		X			
LAMP2	Lysosomal-associated membrane protein 2	Involving in the process of the autophagosome maturation in lysosomes	XLD	X	X			
LDB3	LIM domain binding 3	Providing structural stability to the Z-disc	AD	X	X			
LMNA	Lamin A/C	Component of the nuclear envelope; implicating in cell cycle regulation, chromatin organization and DNA damage response	AD, AR		X		X	X
MYBPC3	Myosin-binding protein C	Linking the thin and thick filaments of the sarcomeres	AD, AR	X	X			X
MYH6	Alpha myosin heavy chain 6	Component of the cardiac myosin, which makes up the thick filaments of the sarcomeres	AD	X	X			
MYH7	Beta myosin heavy chain 7	Major component of the cardiac myosin, which makes up the thick filaments of the sarcomeres	AD, DD, AR	X	X	X		X
MYL2	Myosin regulatory light chain 2	Essential for the activation of the cardiac myosin during muscle contraction	AD	X		X		
MYL3	Myosin essential light chain 3	Stabilizing the alpha-helical neck of the cardiac myosin head	AD, AR	X		X		
MYLK2	Myosin light chain kinase 2	Phosphorylating the light chains of myosin, thereby regulating the actin-myosin interaction	AD, DD	X				
MYOM1	Myomesin 1	Structural and functional role in the M-band of the sarcomeres	N/A	X				
MYOZ2	Myozenin 2	Tethering calcineurin to alpha-actinin of the sarcomeres, thus it	AD	X				

		is important for calcineurin signaling						
MYPN	Myopalladin	Part of the alpha-actinin-myopalladin-nebulette complex that tethers titin and actin to the Z-disc of the sarcomeres	AD, AR		X	X		
NEBL	Nebulette	Aligning the thin filaments of the sarcomeres and linking them to the Z-disc	N/A		X			
NEXN	Nexilin	Involving in Z-disc stability	AD	X	X			
NKX2.5	NK2 homeobox 5	Homeobox-containing transcription factor, which is important for normal cardiac development	AD	X				
PDLIM3	PDZ and LIM domain protein 3	Involving in cytoskeletal assembly	N/A		X			
PKP2	Plakophilin 2	Component of the desmosome, which provides adhesion between cardiac cells	AD		X		X	
PLN	Phospholamban	Playing key role in the release and storage of calcium in the sarcoplasmic reticulum	AD	X	X		X	
PRDM16	PR domain containing 16	Transcription factor, which is important for normal cardiac development	AD		X			X
PRKAG2	AMP-activated protein kinase, non-catalytic subunit gamma 2	Involving in cellular ATP metabolic regulation	AD	X				
PTPN11	Protein tyrosine phosphatase SHP2	Involving in the regulation of RAS/MAPK signalling pathway, which regulates important cell functions (proliferation, differentiation, migration, apoptosis); critical in normal cardiac development	AD	X				

RAF1	Raf-1 proto-oncogene, serine/threonine kinase	Part of the RAS/MAPK signalling pathway, which regulates important cell functions (proliferation, differentiation, migration, apoptosis)	AD	X				
RBM20	RNA-binding motif protein 20	Binding RNA and regulating mRNA splicing of genes involved in cardiac development	AD		X			
RYR2	Ryanodine receptor 2	Making up calcium channels, which control the flow of calcium ions through the sarcoplasmic reticulum	AD	X			X	
SCN5A	Voltage-gated sodium channel subunit alpha Nav1.5	Making up sodium channels, which control the flow of sodium ions into cardiomyocytes	AD, AR		X		X	
SGCD	Sarcoglycan delta	Component of the sarcoglycan complex, which is a member of dystrophin-glycoprotein complex, which bridges the cytoskeleton and the extracellular matrix	AD?, AR		X			
TAZ	Tafazzin	Involving in the metabolism of cardiolipin, the main phospholipid of the mitochondrial inner membrane	XLR	X	X			X
TCAP	Titin-cap protein	Involving in sarcomere development and stability	AD, AR	X	X			
TGFB3	Transforming growth factor beta 3	Binding to receptor proteins on the surface of cells and triggering the cell signalling within the cells, which regulates important cell functions (proliferation, differentiation, migration, apoptosis)	AD				X	
TMEM43	Transmembrane protein 43	Involving in the organization of the nuclear membrane	AD				X	

TMPO	Thymopoietin	Maintaining the structural organization of the nuclear envelope	N/A		X			
TNNC1	Troponin C type 1	Acting as a myofilament calcium sensor; the binding of calcium to troponin C allows the interaction of actin with myosin for the generation of contractile force	AD	X	X			
TNNI3	Troponin I type 3	The inhibitory protein of the troponin complex; blocking the actin-myosin interaction in response to the reduction in the intracellular calcium concentration, leading to the muscle relaxation	AD, AR	X	X	X		
TNNT2	Troponin T type 2	The tropomyosin-binding subunit of the troponin complex, which regulates the muscle contraction in response to the changes of the intracellular calcium concentration	AD	X	X	X		X
TPM1	Tropomyosin 1	Maintaining the stability of the thin filament of the sarcomeres; regulating the calcium-dependent actin-myosin interaction	AD	X	X	X		X
TTN	Titin	Maintaining sarcomere structural integrity	AD, AR	X	X	X	X	
TTR	Transthyretin	Involving in the transportation of hormone thyroxine and retinol (vitamin A) throughout the body	AD	X	X	X		X
VCL	Vinculin	Involving in the anchoring of F-actin to the sarcolemma, thus it is important for cell-cell and cell-extracellular matrix junctions	AD	X	X			X

AD: Autosomal Dominant, AR: Autosomal Recessive, DD: Digenic Dominant, XL: X-linked, XLD: X-linked Dominant, XLR: X-linked Recessive

Table S2: Variants which based on our data were upgraded to Likely Pathogenic and description of the relevant ACMG criteria.

Family	Gene-Exon	Chromosome position; Transcript	Coding	Protein	Franklin classification	Cosegregation data	Our classification
FAM03 (HOCM)	*TNNI3* (Exon 7)	chr19:55665519; NM_000363.4	c.428C>A	p.Thr143Asn	VUS to likely pathogenic (PM1: Pathogenic Moderate, PP2: Pathogenic Supporting, PM2: Pathogenic Moderate)	Proband with HOCM carries the variant, whereas his healthy son does not	Likely pathogenic
FAM10 (HOCM)	*PLN* (Exon 2)	chr6:118880229; NM_002667.4	c.145G>A	p.Val49Met	VUS to likely pathogenic (PM2: Pathogenic Moderate, PP3: Pathogenic Moderate)	Proband with HOCM carries the variant, whereas the healthy family members do not. His mother and his grandmother, who have milder symptoms, carry also the variant.	Likely pathogenic
FAM24 (ARVC)	*DSC2* (Exon 8)	chr18:28662978; NM_024422.4	c.991C>A	p.Gln331Lys	VUS (PM2: Pathogenic Moderate)	Proband with ARVC carries the variant as well as his brothers with ARVC. His father, who is healthy, does not carry the variant.	Likely pathogenic

CAD: Coronary Artery Disease, HOCM: Hypertrophic Obstructive Cardiomyopathy, ICD: Implantable Cardioverter Defibrillator, ISH: Isolated Septal Hypertrophy

FAM01

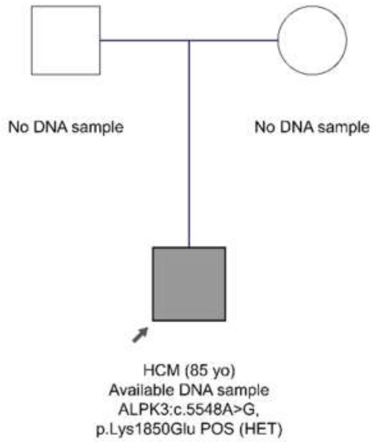

FAM02

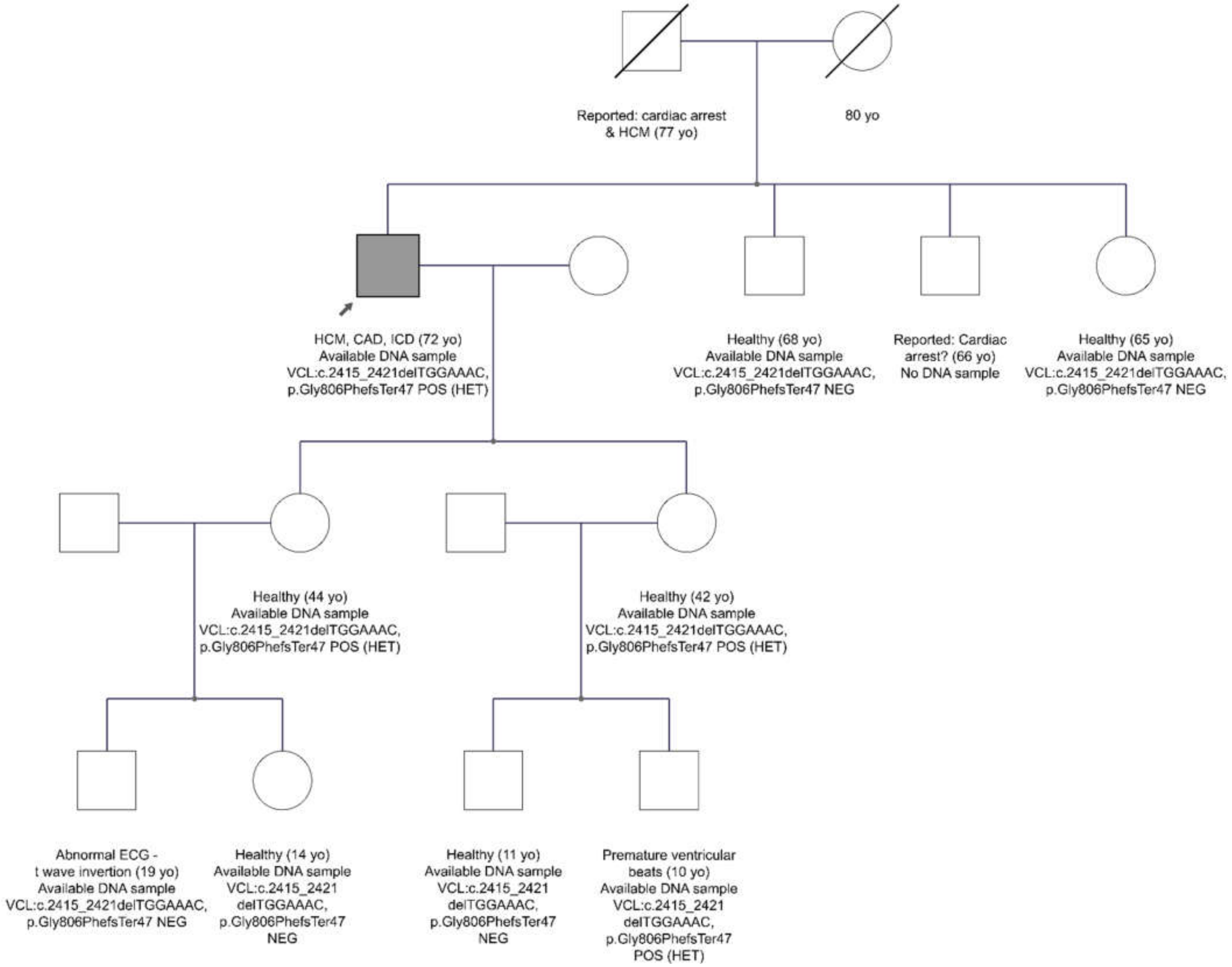

FAM03

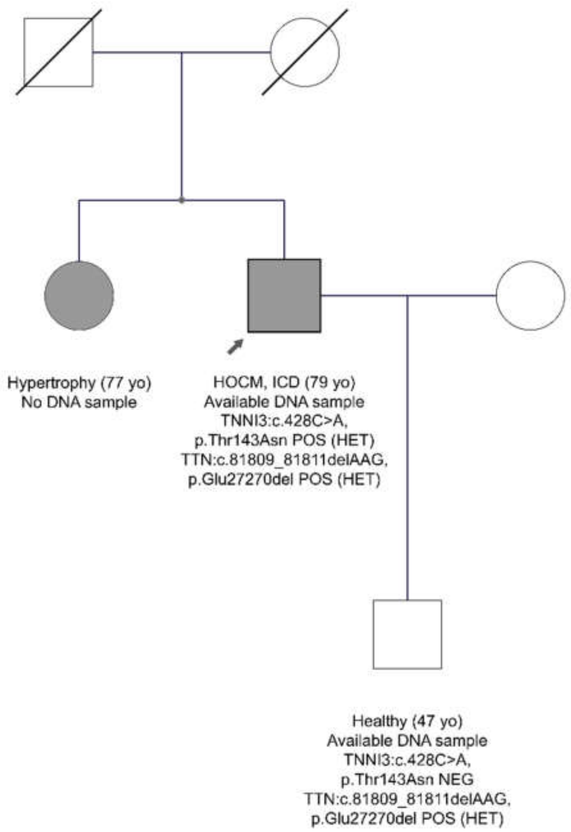

Hypertrophy (77 yo)
No DNA sample
HOCM, ICD (79 yo)
Available DNA sample
TNNI3:c.428C>A,
p.Thr143Asn POS (HET)
TTN:c.81809_81811delAAG,
p.Glu27270del POS (HET)
Healthy (47 yo)
Available DNA sample
TNNI3:c.428C>A,
p.Thr143Asn NEG
TTN:c.81809_81811delAAG,
p.Glu27270del POS (HET)

FAM04

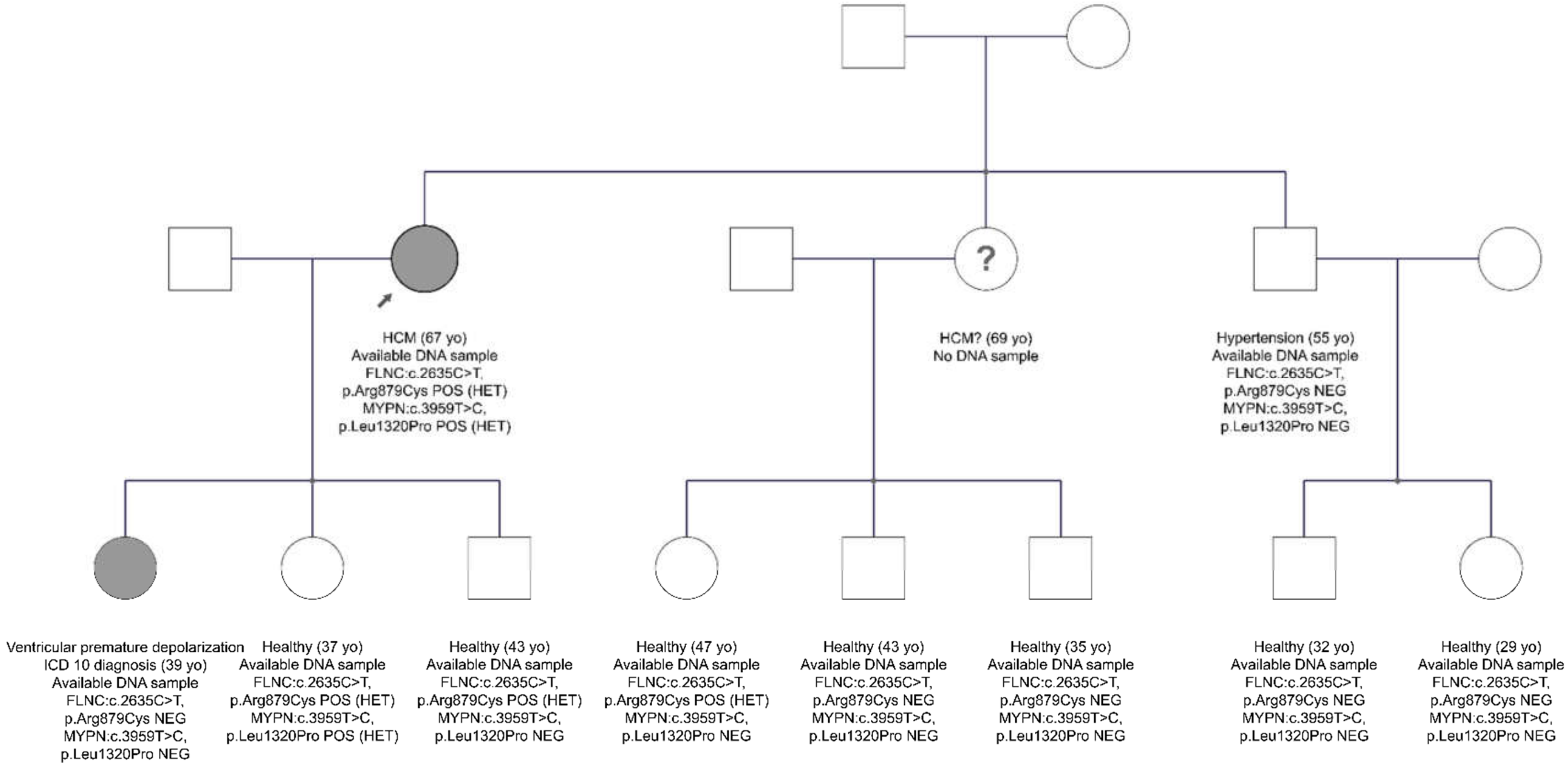

HCM (67 yo)
Available DNA sample
FLNC:c.2635C>T,
p.Arg879Cys POS (HET)
MYPN:c.3959T>C,
p.Leu1320Pro POS (HET)
?
HCM? (69 yo)
No DNA sample
Hypertension (55 yo)
Available DNA sample
FLNC:c.2635C>T,
p.Arg879Cys NEG
MYPN:c.3959T>C,
p.Leu1320Pro NEG
Ventricular premature depolarization
ICD 10 diagnosis (39 yo)
Available DNA sample
FLNC:c.2635C>T,
p.Arg879Cys NEG
MYPN:c.3959T>C,
p.Leu1320Pro NEG
Healthy (37 yo)
Available DNA sample
FLNC:c.2635C>T,
p.Arg879Cys POS (HET)
MYPN:c.3959T>C,
p.Leu1320Pro POS (HET)
Healthy (43 yo)
Available DNA sample
FLNC:c.2635C>T,
p.Arg879Cys POS (HET)
MYPN:c.3959T>C,
p.Leu1320Pro NEG
Healthy (47 yo)
Available DNA sample
FLNC:c.2635C>T,
p.Arg879Cys POS (HET)
MYPN:c.3959T>C,
p.Leu1320Pro NEG
Healthy (43 yo)
Available DNA sample
FLNC:c.2635C>T,
p.Arg879Cys NEG
MYPN:c.3959T>C,
p.Leu1320Pro NEG
Healthy (35 yo)
Available DNA sample
FLNC:c.2635C>T,
p.Arg879Cys NEG
MYPN:c.3959T>C,
p.Leu1320Pro NEG
Healthy (32 yo)
Available DNA sample
FLNC:c.2635C>T,
p.Arg879Cys NEG
MYPN:c.3959T>C,
p.Leu1320Pro NEG
Healthy (29 yo)
Available DNA sample
FLNC:c.2635C>T,
p.Arg879Cys NEG
MYPN:c.3959T>C,
p.Leu1320Pro NEG

FAM05

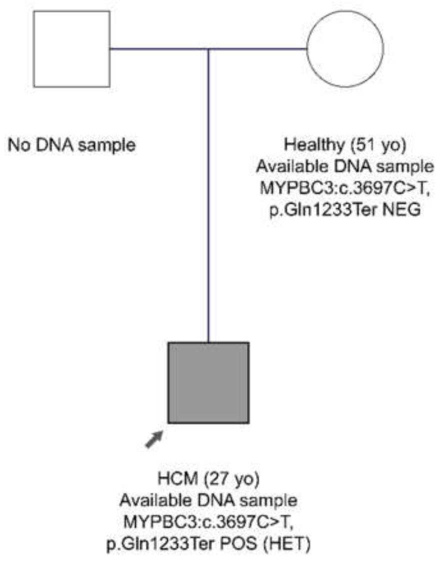
No DNA sample
Healthy (51 yo)
Available DNA sample
MYPBC3:c.3697C>T,
p.Gln1233Ter NEG
HCM (27 yo)
Available DNA sample
MYPBC3:c.3697C>T,
p.Gln1233Ter POS (HET)

FAM06

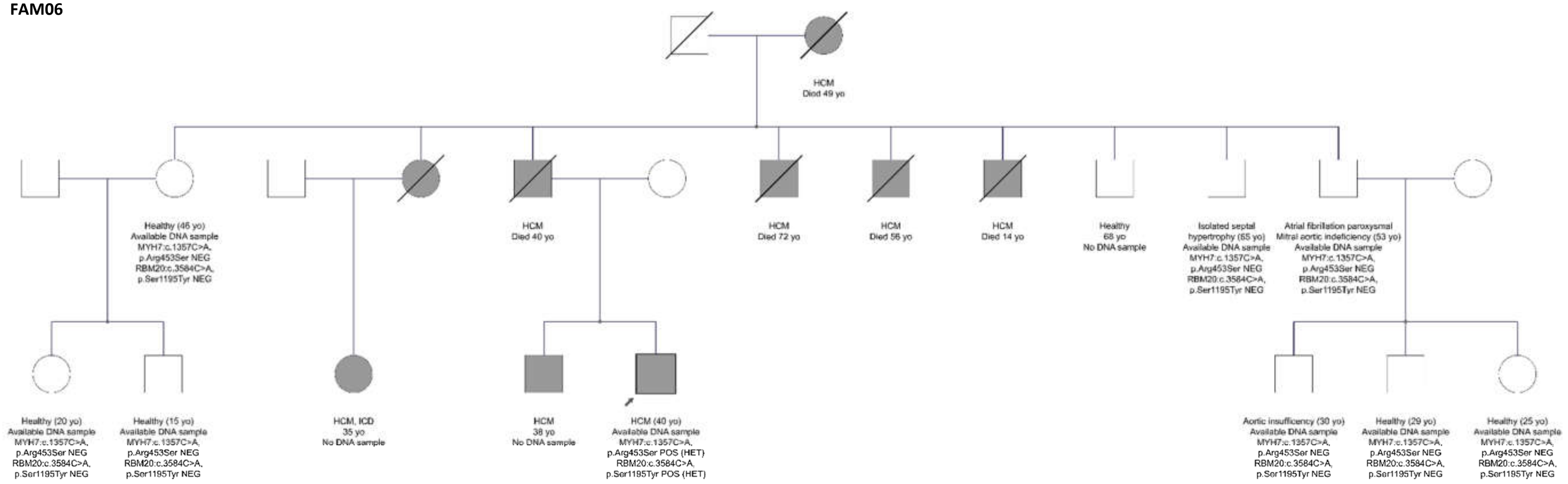
HCM
Died 49 yo
Healthy (46 yo)
Available DNA sample
MYH7:c.1357C>A,
p.Arg453Ser NEG
RBM20:c.3584C>A,
p.Ser1195Tyr NEG
HCM
Died 40 yo
HCM
Died 72 yo
HCM
Died 56 yo
HCM
Died 14 yo
Healthy
68 yo
No DNA sample
Isolated septal
hypertrophy (65 yo)
Available DNA sample
MYH7:c.1357C>A,
p.Arg453Ser NEG
RBM20:c.3584C>A,
p.Ser1195Tyr NEG
Atrial fibrillation paroxysmal
Mitral aortic indeficiency (53 yo)
Available DNA sample
MYH7:c.1357C>A,
p.Arg453Ser NEG
RBM20:c.3584C>A,
p.Ser1195Tyr NEG
Healthy (20 yo)
Available DNA sample
MYH7:c.1357C>A,
p.Arg453Ser NEG
RBM20:c.3584C>A,
p.Ser1195Tyr NEG
Healthy (15 yo)
Available DNA sample
MYH7:c.1357C>A,
p.Arg453Ser NEG
RBM20:c.3584C>A,
p.Ser1195Tyr NEG
HCM, ICD
35 yo
No DNA sample
HCM
38 yo
No DNA sample
HCM (40 yo)
Available DNA sample
MYH7:c.1357C>A,
p.Arg453Ser POS (HET)
RBM20:c.3584C>A,
p.Ser1195Tyr POS (HET)
Aortic insufficency (30 yo)
Available DNA sample
MYH7:c.1357C>A,
p.Arg453Ser NEG
RBM20:c.3584C>A,
p.Ser1195Tyr NEG
Healthy (29 yo)
Available DNA sample
MYH7:c.1357C>A,
p.Arg453Ser NEG
RBM20:c.3584C>A,
p.Ser1195Tyr NEG
Healthy (25 yo)
Available DNA sample
MYH7:c.1357C>A,
p.Arg453Ser NEG
RBM20:c.3584C>A,
p.Ser1195Tyr NEG

FAM07

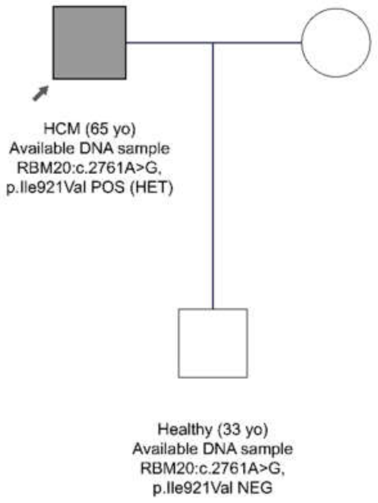
HCM (65 yo)
Available DNA sample
RBM20:c.2761A>G,
p.Ile921Val POS (HET)
Healthy (33 yo)
Available DNA sample
RBM20:c.2761A>G,
p.Ile921Val NEG

FAM08

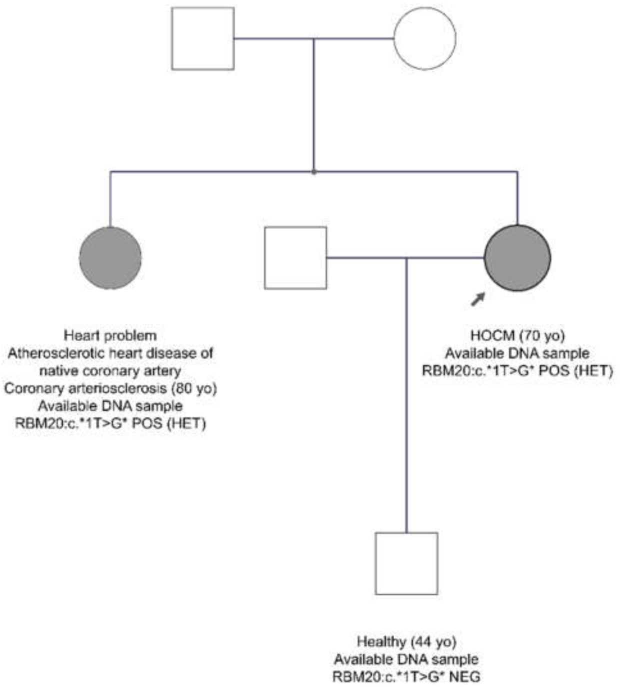
Heart problem
Atherosclerotic heart disease of
native coronary artery
Coronary arteriosclerosis (80 yo)
Available DNA sample
RBM20:c.*1T>G* POS (HET)
HOCM (70 yo)
Available DNA sample
RBM20:c.*1T>G* POS (HET)
Healthy (44 yo)
Available DNA sample
RBM20:c.*1T>G* NEG

FAM09

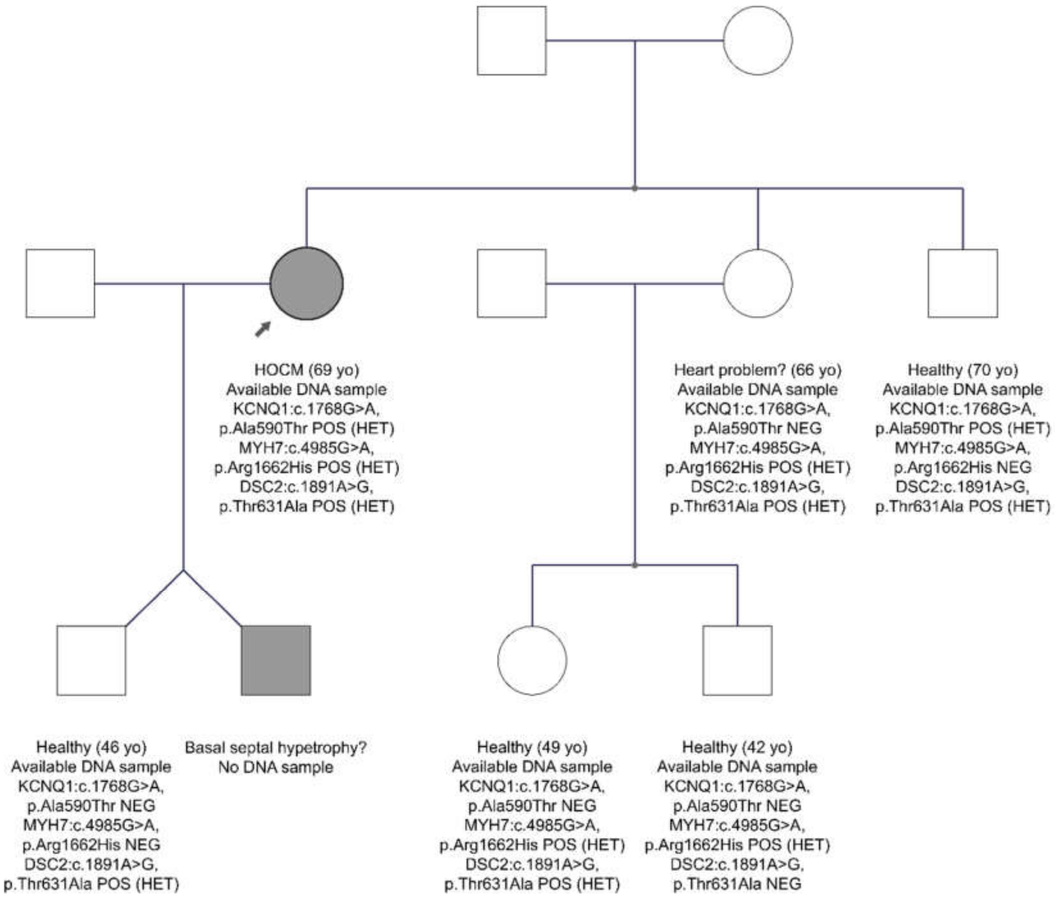
HOCM (69 yo)
Available DNA sample
KCNQ1:c.1768G>A,
p.Ala590Thr POS (HET)
MYH7:c.4985G>A,
p.Arg1662His POS (HET)
DSC2:c.1891A>G,
p.Thr631Ala POS (HET)
Heart problem? (66 yo)
Available DNA sample
KCNQ1:c.1768G>A,
p.Ala590Thr NEG
MYH7:c.4985G>A,
p.Arg1662His POS (HET)
DSC2:c.1891A>G,
p.Thr631Ala POS (HET)
Healthy (70 yo)
Available DNA sample
KCNQ1:c.1768G>A,
p.Ala590Thr POS (HET)
MYH7:c.4985G>A,
p.Arg1662His NEG
DSC2:c.1891A>G,
p.Thr631Ala POS (HET)
Healthy (46 yo)
Available DNA sample
KCNQ1:c.1768G>A,
p.Ala590Thr NEG
MYH7:c.4985G>A,
p.Arg1662His NEG
DSC2:c.1891A>G,
p.Thr631Ala POS (HET)
Basal septal hypetrophy?
No DNA sample
Healthy (49 yo)
Available DNA sample
KCNQ1:c.1768G>A,
p.Ala590Thr NEG
MYH7:c.4985G>A,
p.Arg1662His POS (HET)
DSC2:c.1891A>G,
p.Thr631Ala POS (HET)
Healthy (42 yo)
Available DNA sample
KCNQ1:c.1768G>A,
p.Ala590Thr NEG
MYH7:c.4985G>A,
p.Arg1662His POS (HET)
DSC2:c.1891A>G,
p.Thr631Ala NEG

FAM10

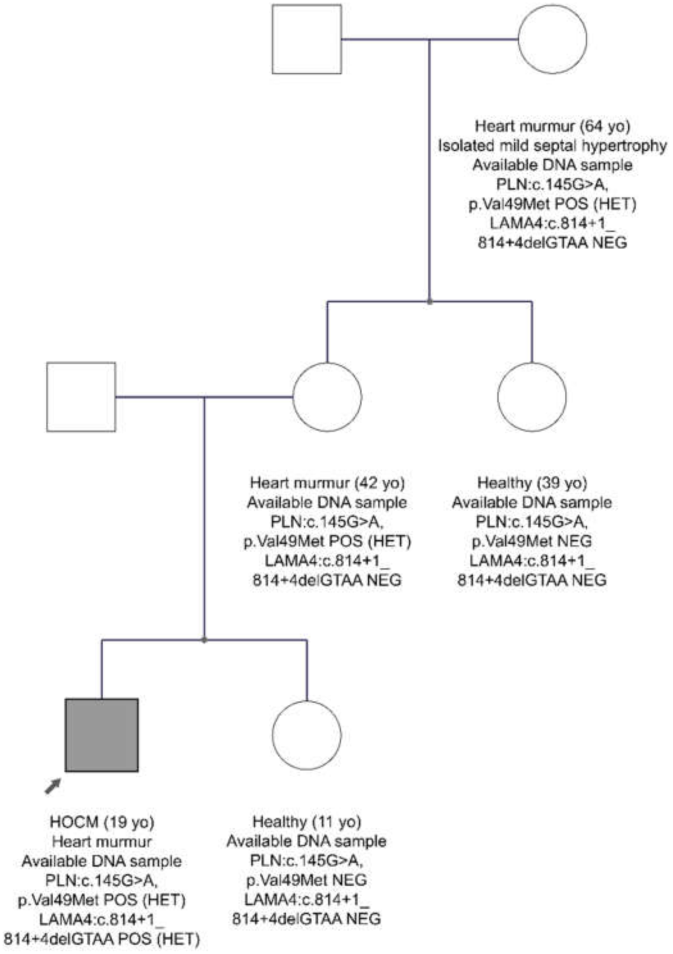
Heart murmur (64 yo)
Isolated mild septal hypertrophy
Available DNA sample
PLN:c.145G>A,
p.Val49Met POS (HET)
LAMA4:c.814+1_
814+4delGTAA NEG
Heart murmur (42 yo)
Available DNA sample
PLN:c.145G>A,
p.Val49Met POS (HET)
LAMA4:c.814+1_
814+4delGTAA NEG
Healthy (39 yo)
Available DNA sample
PLN:c.145G>A,
p.Val49Met NEG
LAMA4:c.814+1_
814+4delGTAA NEG
HOCM (19 yo)
Heart murmur
Available DNA sample
PLN:c.145G>A,
p.Val49Met POS (HET)
LAMA4:c.814+1_
814+4delGTAA POS (HET)
Healthy (11 yo)
Available DNA sample
PLN:c.145G>A,
p.Val49Met NEG
LAMA4:c.814+1_
814+4delGTAA NEG

FAM11

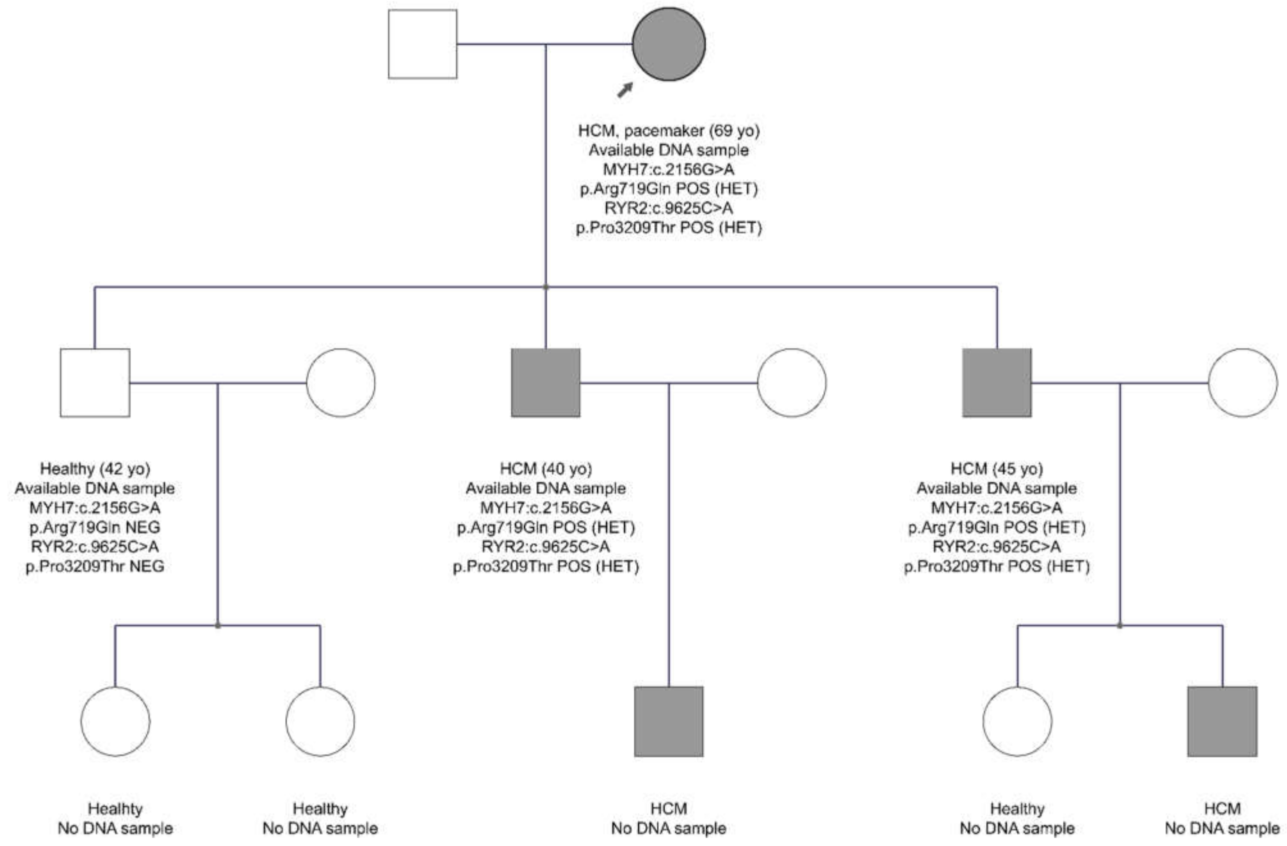
HCM, pacemaker (69 yo)
Available DNA sample
MYH7:c.2156G>A
p.Arg719Gln POS (HET)
RYR2:c.9625C>A
p.Pro3209Thr POS (HET)
Healthy (42 yo)
Available DNA sample
MYH7:c.2156G>A
p.Arg719Gln NEG
RYR2:c.9625C>A
p.Pro3209Thr NEG
HCM (40 yo)
Available DNA sample
MYH7:c.2156G>A
p.Arg719Gln POS (HET)
RYR2:c.9625C>A
p.Pro3209Thr POS (HET)
HCM (45 yo)
Available DNA sample
MYH7:c.2156G>A
p.Arg719Gln POS (HET)
RYR2:c.9625C>A
p.Pro3209Thr POS (HET)
Healhty
No DNA sample
Healthy
No DNA sample
HCM
No DNA sample
Healthy
No DNA sample
HCM
No DNA sample

FAM12

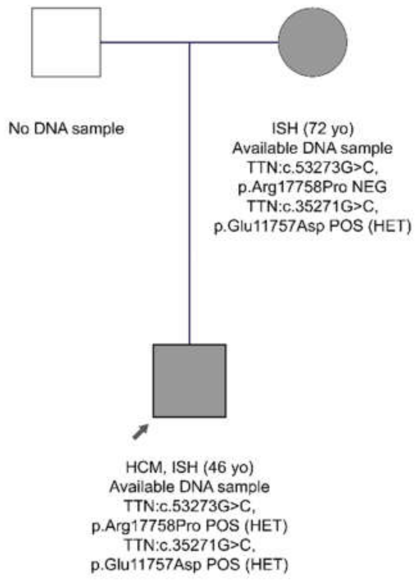
No DNA sample
ISH (72 yo)
Available DNA sample
TTN:c.53273G>C,
p.Arg17758Pro NEG
TTN:c.35271G>C,
p.Glu11757Asp POS (HET)
HCM, ISH (46 yo)
Available DNA sample
TTN:c.53273G>C,
p.Arg17758Pro POS (HET)
TTN:c.35271G>C,
p.Glu11757Asp POS (HET)

FAM13

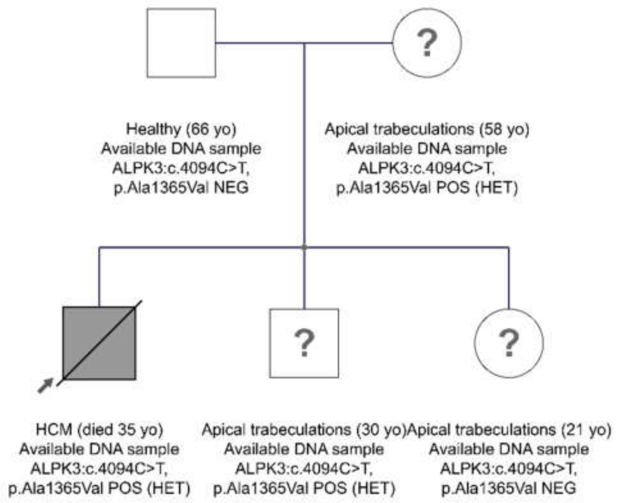
?
Healthy (66 yo)
Available DNA sample
ALPK3:c.4094C>T,
p.Ala1365Val NEG
Apical trabeculations (58 yo)
Available DNA sample
ALPK3:c.4094C>T,
p.Ala1365Val POS (HET)
?
?
HCM (died 35 yo)
Available DNA sample
ALPK3:c.4094C>T,
p.Ala1365Val POS (HET)
Apical trabeculations (30 yo)
Available DNA sample
ALPK3:c.4094C>T,
p.Ala1365Val POS (HET)
Apical trabeculations (21 yo)
Available DNA sample
ALPK3:c.4094C>T,
p.Ala1365Val NEG

FAM14

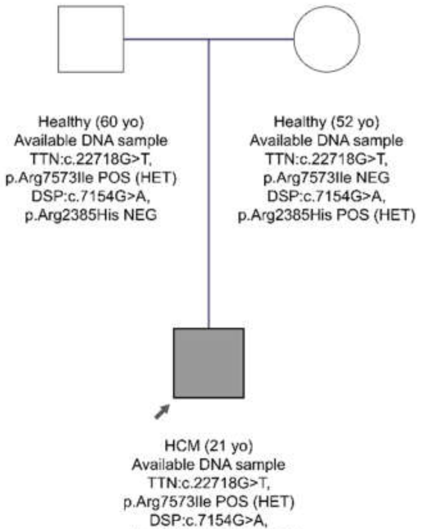
Healthy (60 yo)
Available DNA sample
TTN:c.22718G>T,
p.Arg7573Ile POS (HET)
DSP:c.7154G>A,
p.Arg2385His NEG
Healthy (52 yo)
Available DNA sample
TTN:c.22718G>T,
p.Arg7573Ile NEG
DSP:c.7154G>A,
p.Arg2385His POS (HET)
HCM (21 yo)
Available DNA sample
TTN:c.22718G>T,
p.Arg7573Ile POS (HET)
DSP:c.7154G>A,

FAM15

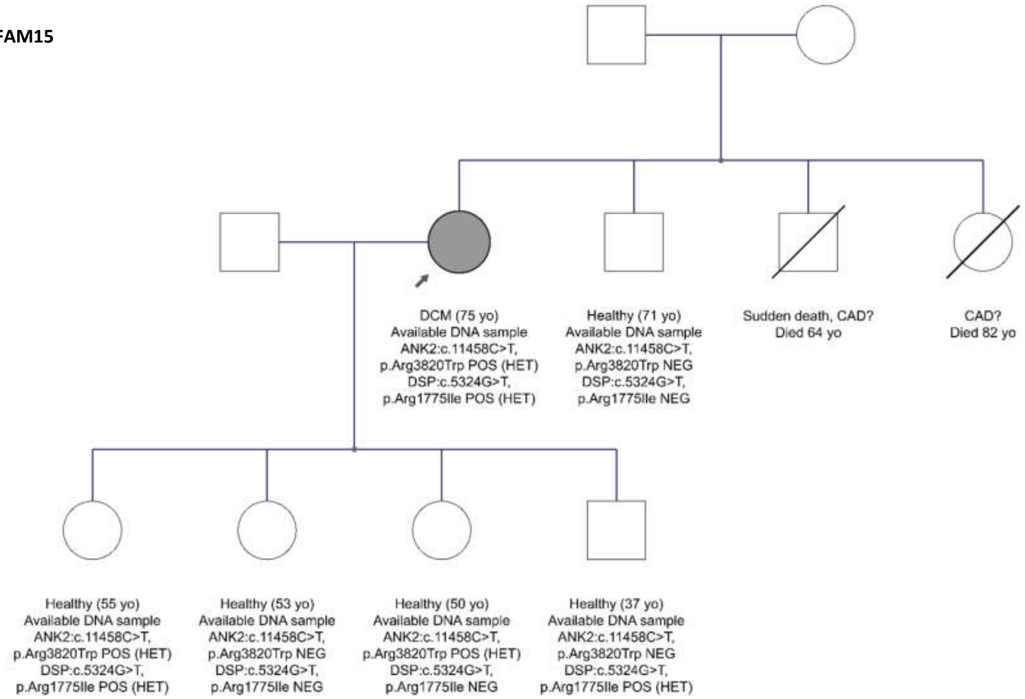
DCM (75 yo)
Available DNA sample
ANK2:c.11458C>T,
p.Arg3820Trp POS (HET)
DSP:c.5324G>T,
p.Arg1775Ile POS (HET)
Healthy (71 yo)
Available DNA sample
ANK2:c.11458C>T,
p.Arg3820Trp NEG
DSP:c.5324G>T,
p.Arg1775Ile NEG
Sudden death, CAD?
Died 64 yo
CAD?
Died 82 yo
Healthy (55 yo)
Available DNA sample
ANK2:c.11458C>T,
p.Arg3820Trp POS (HET)
DSP:c.5324G>T,
p.Arg1775Ile POS (HET)
Healthy (53 yo)
Available DNA sample
ANK2:c.11458C>T,
p.Arg3820Trp NEG
DSP:c.5324G>T,
p.Arg1775Ile NEG
Healthy (50 yo)
Available DNA sample
ANK2:c.11458C>T,
p.Arg3820Trp POS (HET)
DSP:c.5324G>T,
p.Arg1775Ile NEG
Healthy (37 yo)
Available DNA sample
ANK2:c.11458C>T,
p.Arg3820Trp NEG
DSP:c.5324G>T,
p.Arg1775Ile POS (HET)

FAM16

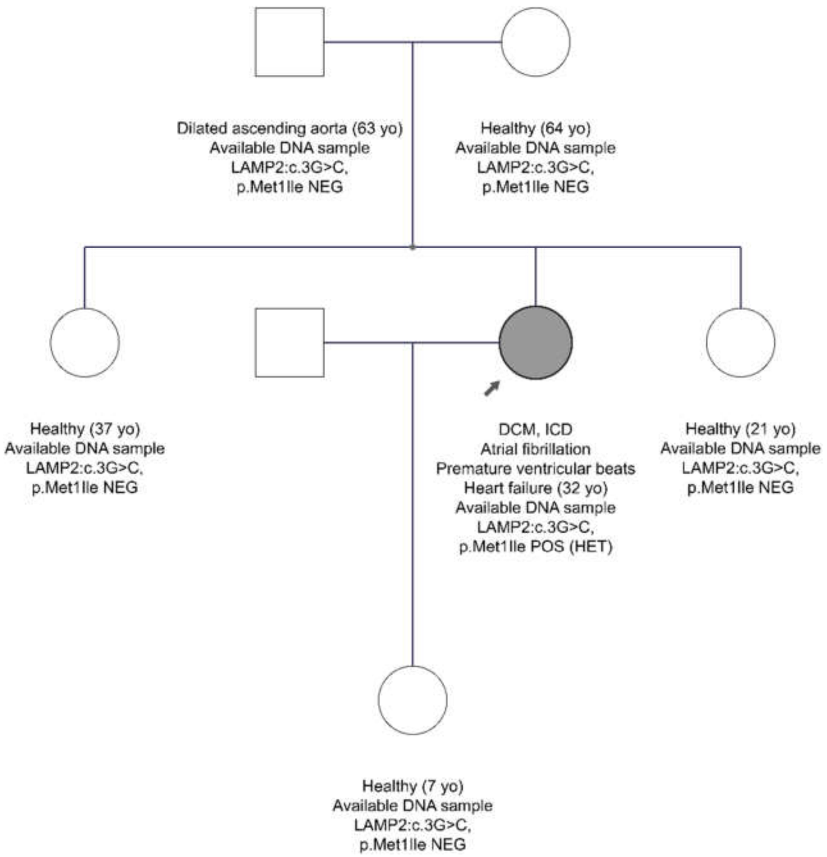
Dilated ascending aorta (63 yo)
Available DNA sample
LAMP2:c.3G>C,
p.Met1Ile NEG
Healthy (64 yo)
Available DNA sample
LAMP2:c.3G>C,
p.Met1Ile NEG
Healthy (37 yo)
Available DNA sample
LAMP2:c.3G>C,
p.Met1Ile NEG
DCM, ICD
Atrial fibrillation
Premature ventricular beats
Heart failure (32 yo)
Available DNA sample
LAMP2:c.3G>C,
p.Met1Ile POS (HET)
Healthy (21 yo)
Available DNA sample
LAMP2:c.3G>C,
p.Met1Ile NEG
Healthy (7 yo)
Available DNA sample
LAMP2:c.3G>C,
p.Met1Ile NEG

FAM17

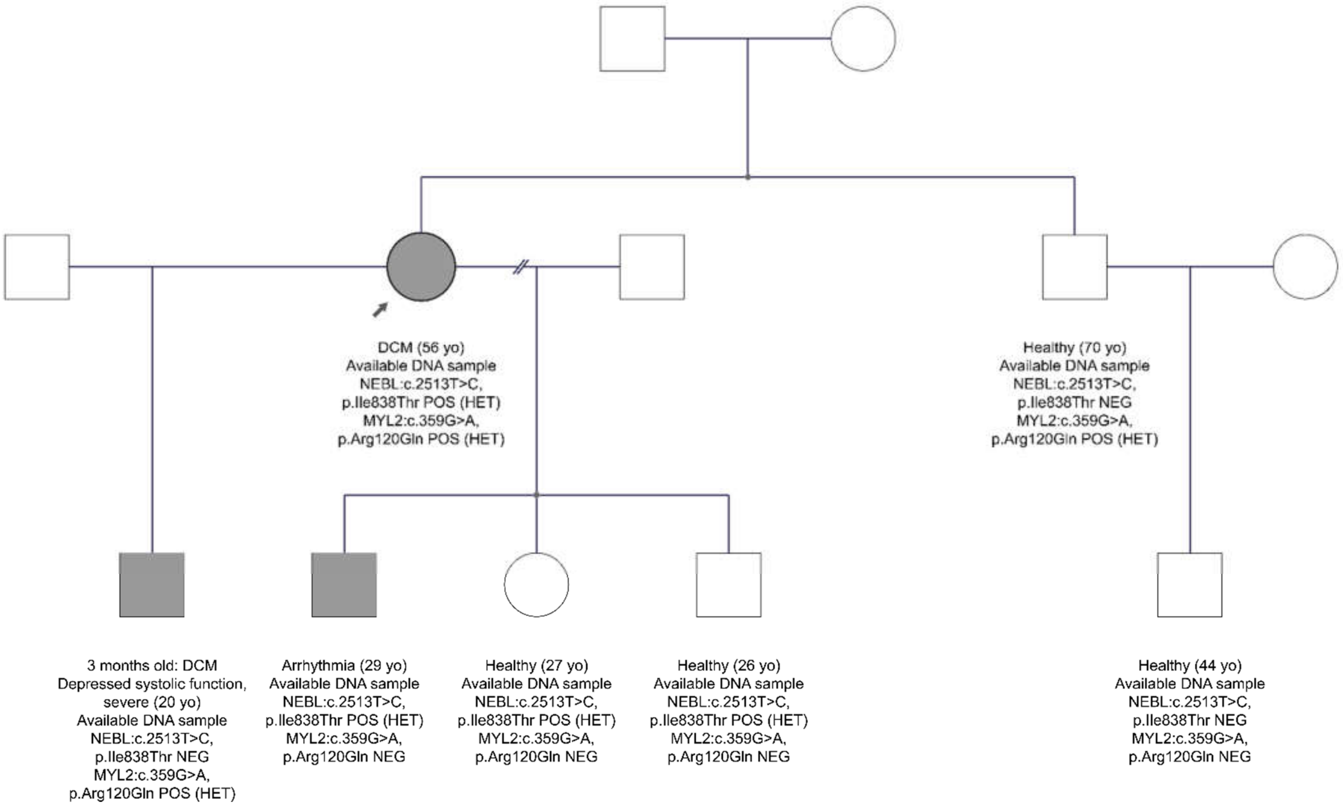
DCM (56 yo)
Available DNA sample
NEBL:c.2513T>C,
p.Ile838Thr POS (HET)
MYL2:c.359G>A,
p.Arg120Gln POS (HET)
Healthy (70 yo)
Available DNA sample
NEBL:c.2513T>C,
p.Ile838Thr NEG
MYL2:c.359G>A,
p.Arg120Gln POS (HET)
3 months old: DCM
Depressed systolic function,
severe (20 yo)
Available DNA sample
NEBL:c.2513T>C,
p.Ile838Thr NEG
MYL2:c.359G>A,
p.Arg120Gln POS (HET)
Arrhythmia (29 yo)
Available DNA sample
NEBL:c.2513T>C,
p.Ile838Thr POS (HET)
MYL2:c.359G>A,
p.Arg120Gln NEG
Healthy (27 yo)
Available DNA sample
NEBL:c.2513T>C,
p.Ile838Thr POS (HET)
MYL2:c.359G>A,
p.Arg120Gln NEG
Healthy (26 yo)
Available DNA sample
NEBL:c.2513T>C,
p.Ile838Thr POS (HET)
MYL2:c.359G>A,
p.Arg120Gln NEG
Healthy (44 yo)
Available DNA sample
NEBL:c.2513T>C,
p.Ile838Thr NEG
MYL2:c.359G>A,
p.Arg120Gln NEG

FAM18

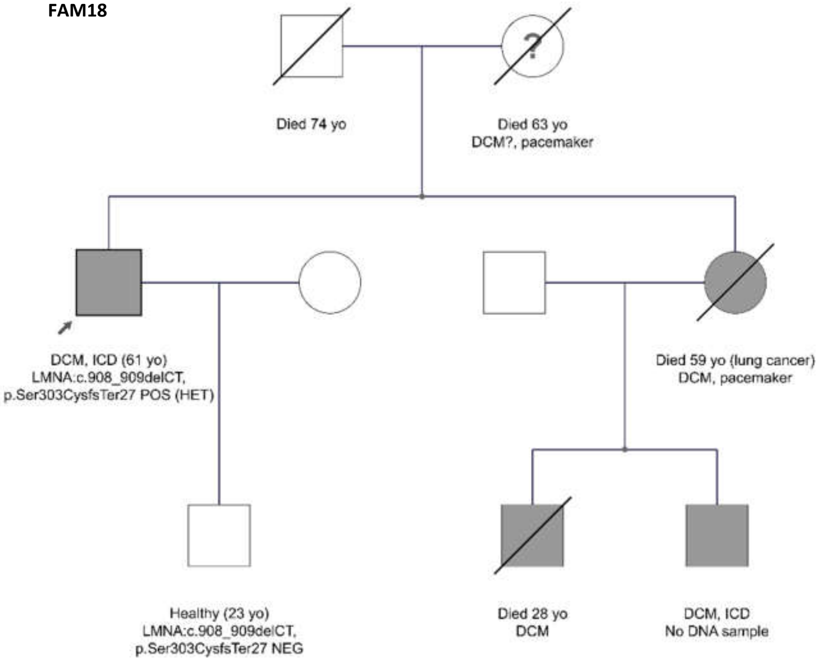
Died 74 yo
Died 63 yo
DCM?, pacemaker
DCM, ICD (61 yo)
LMNA:c.908_909delCT,
p.Ser303CysfsTer27 POS (HET)
Died 59 yo (lung cancer)
DCM, pacemaker
Healthy (23 yo)
LMNA:c.908_909delCT,
p.Ser303CysfsTer27 NEG
Died 28 yo
DCM
DCM, ICD
No DNA sample

FAM19

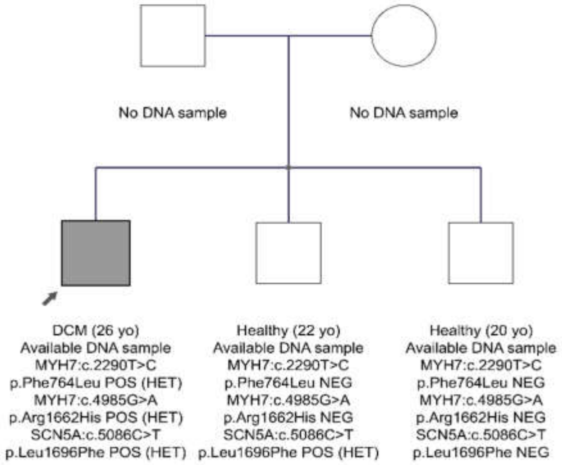
No DNA sample
No DNA sample
DCM (26 yo)
Available DNA sample
MYH7:c.2290T>C
p.Phe764Leu POS (HET)
MYH7:c.4985G>A
p.Arg1662His POS (HET)
SCN5A:c.5086C>T
p.Leu1696Phe POS (HET)
Healthy (22 yo)
Available DNA sample
MYH7:c.2290T>C
p.Phe764Leu NEG
MYH7:c.4985G>A
p.Arg1662His NEG
SCN5A:c.5086C>T
p.Leu1696Phe POS (HET)
Healthy (20 yo)
Available DNA sample
MYH7:c.2290T>C
p.Phe764Leu NEG
MYH7:c.4985G>A
p.Arg1662His NEG
SCN5A:c.5086C>T
p.Leu1696Phe NEG

FAM20

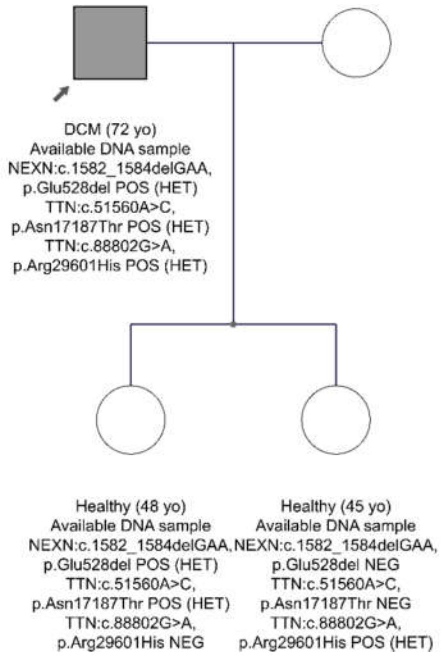
DCM (72 yo)
Available DNA sample
NEXN:c.1582_1584delGAA,
p.Glu528del POS (HET)
TTN:c.51560A>C,
p.Asn17187Thr POS (HET)
TTN:c.88802G>A,
p.Arg29601His POS (HET)
Healthy (48 yo)
Available DNA sample
NEXN:c.1582_1584delGAA,
p.Glu528del POS (HET)
TTN:c.51560A>C,
p.Asn17187Thr POS (HET)
TTN:c.88802G>A,
p.Arg29601His NEG
Healthy (45 yo)
Available DNA sample
NEXN:c.1582_1584delGAA,
p.Glu528del NEG
TTN:c.51560A>C,
p.Asn17187Thr NEG
TTN:c.88802G>A,
p.Arg29601His POS (HET)

FAM21

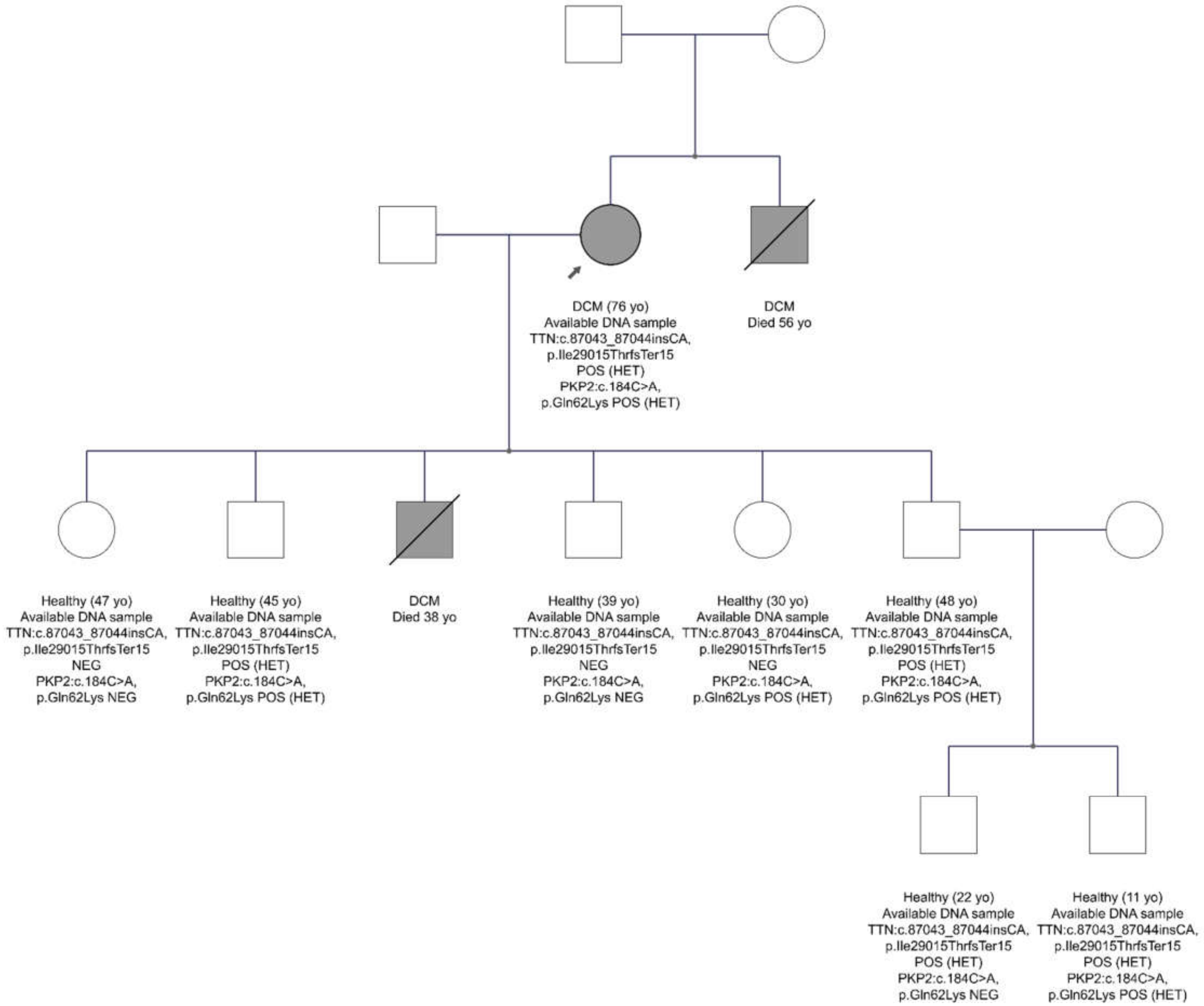

DCM (76 yo)
Available DNA sample
TTN:c.87043_87044insCA,
p.Ile29015ThrfsTer15
POS (HET)
PKP2:c.184C>A,
p.Gln62Lys POS (HET)
DCM
Died 56 yo
Healthy (47 yo)
Available DNA sample
TTN:c.87043_87044insCA,
p.Ile29015ThrfsTer15
NEG
PKP2:c.184C>A,
p.Gln62Lys NEG
Healthy (45 yo)
Available DNA sample
TTN:c.87043_87044insCA,
p.Ile29015ThrfsTer15
POS (HET)
PKP2:c.184C>A,
p.Gln62Lys POS (HET)
DCM
Died 38 yo
Healthy (39 yo)
Available DNA sample
TTN:c.87043_87044insCA,
p.Ile29015ThrfsTer15
NEG
PKP2:c.184C>A,
p.Gln62Lys NEG
Healthy (30 yo)
Available DNA sample
TTN:c.87043_87044insCA,
p.Ile29015ThrfsTer15
NEG
PKP2:c.184C>A,
p.Gln62Lys POS (HET)
Healthy (48 yo)
Available DNA sample
TTN:c.87043_87044insCA,
p.Ile29015ThrfsTer15
POS (HET)
PKP2:c.184C>A,
p.Gln62Lys POS (HET)
Healthy (22 yo)
Available DNA sample
TTN:c.87043_87044insCA,
p.Ile29015ThrfsTer15
POS (HET)
PKP2:c.184C>A,
p.Gln62Lys NEG
Healthy (11 yo)
Available DNA sample
TTN:c.87043_87044insCA,
p.Ile29015ThrfsTer15
POS (HET)
PKP2:c.184C>A,
p.Gln62Lys POS (HET)

FAM22

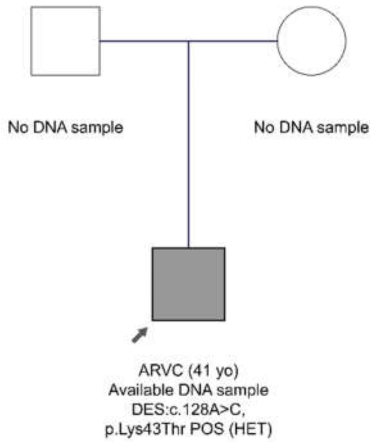

No DNA sample
No DNA sample
ARVC (41 yo)
Available DNA sample
DES:c.128A>C,
p.Lys43Thr POS (HET)

FAM23

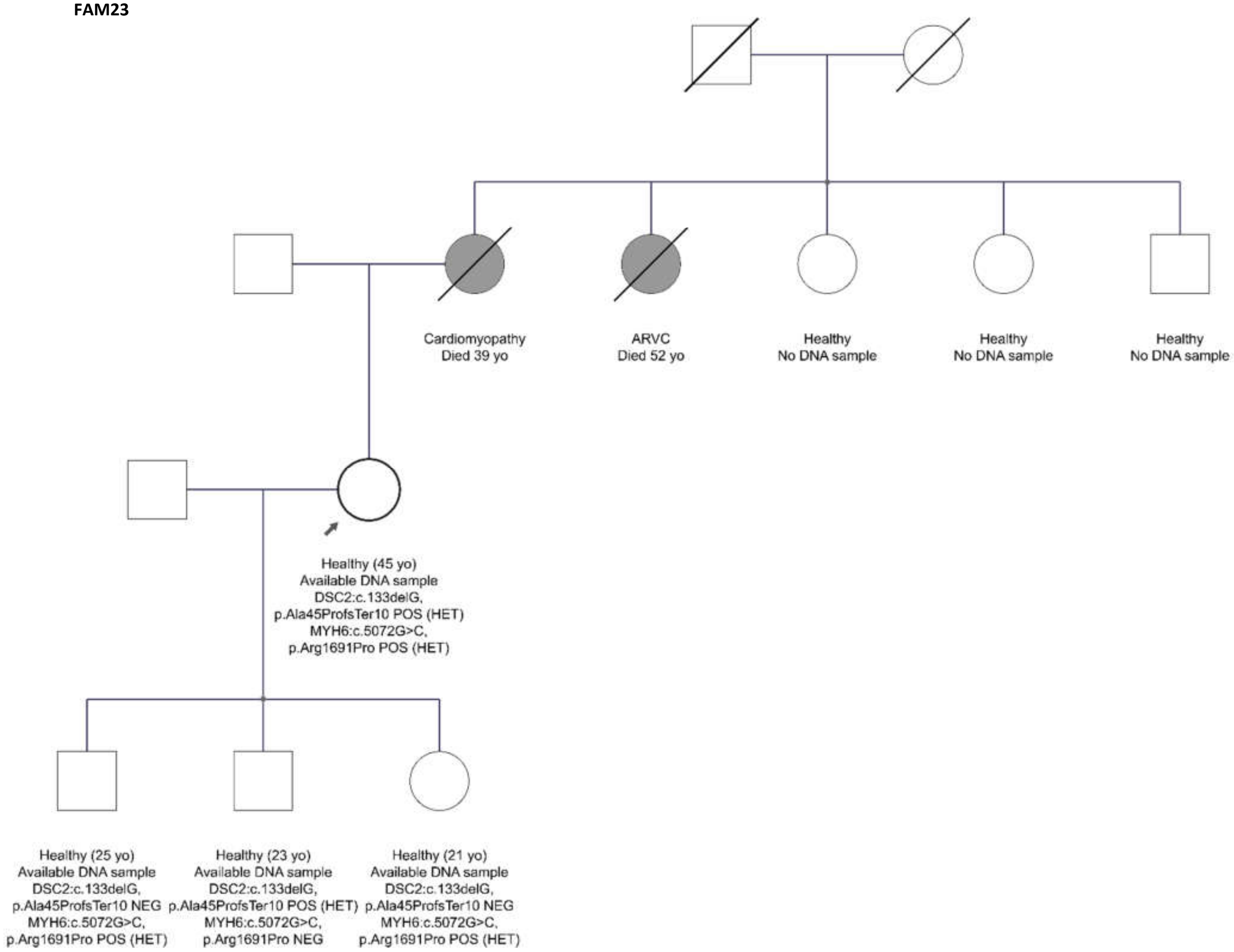

Cardiomyopathy
Died 39 yo
ARVC
Died 52 yo
Healthy
No DNA sample
Healthy
No DNA sample
Healthy
No DNA sample
Healthy (45 yo)
Available DNA sample
DSC2:c.133delG,
p.Ala45ProfsTer10 POS (HET)
MYH6:c.5072G>C,
p.Arg1691Pro POS (HET)
Healthy (25 yo)
Available DNA sample
DSC2:c.133delG,
p.Ala45ProfsTer10 NEG
MYH6:c.5072G>C,
p.Arg1691Pro POS (HET)
Healthy (23 yo)
Available DNA sample
DSC2:c.133delG,
p.Ala45ProfsTer10 POS (HET)
MYH6:c.5072G>C,
p.Arg1691Pro NEG
Healthy (21 yo)
Available DNA sample
DSC2:c.133delG,
p.Ala45ProfsTer10 NEG
MYH6:c.5072G>C,
p.Arg1691Pro POS (HET)

FAM2

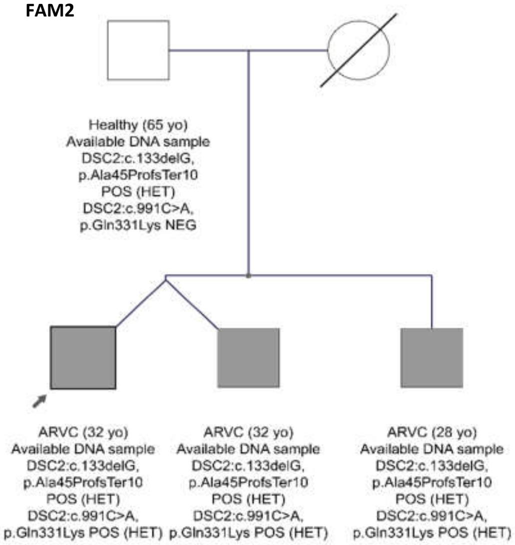
Healthy (65 yo)
Available DNA sample
DSC2:c.133delG,
p.Ala45ProfsTer10
POS (HET)
DSC2:c.991C>A,
p.Gln331Lys NEG
ARVC (32 yo)
Available DNA sample
DSC2:c.133delG,
p.Ala45ProfsTer10
POS (HET)
DSC2:c.991C>A,
p.Gln331Lys POS (HET)
ARVC (32 yo)
Available DNA sample
DSC2:c.133delG,
p.Ala45ProfsTer10
POS (HET)
DSC2:c.991C>A,
p.Gln331Lys POS (HET)
ARVC (28 yo)
Available DNA sample
DSC2:c.133delG,
p.Ala45ProfsTer10
POS (HET)
DSC2:c.991C>A,
p.Gln331Lys POS (HET)

FAM25

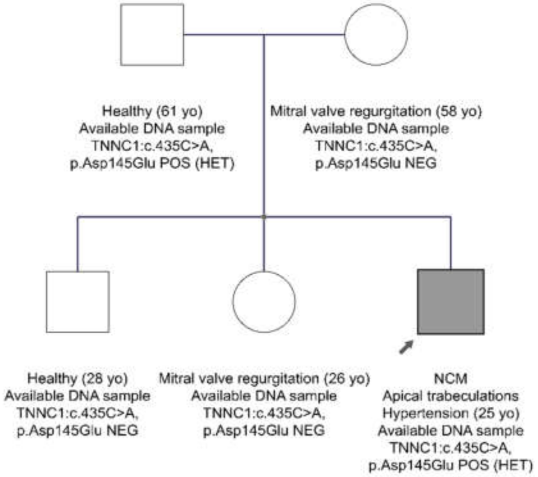
Healthy (61 yo)
Available DNA sample
TNNC1:c.435C>A,
p.Asp145Glu POS (HET)
Mitral valve regurgitation (58 yo)
Available DNA sample
TNNC1:c.435C>A,
p.Asp145Glu NEG
Healthy (28 yo)
Available DNA sample
TNNC1:c.435C>A,
p.Asp145Glu NEG
Mitral valve regurgitation (26 yo)
Available DNA sample
TNNC1:c.435C>A,
p.Asp145Glu NEG
NCM
Apical trabeculations
Hypertension (25 yo)
Available DNA sample
TNNC1:c.435C>A,
p.Asp145Glu POS (HET)

Figure S1: Pedigrees of FAM01-25 described in the paper. The proband is indicated by the black arrow. Information provided below each subject is the clinical data, age, the gene and variant(s) found.
